# Unique DNA methylation signature in HPV-positive head and neck squamous cell carcinomas

**DOI:** 10.1186/s13073-017-0419-z

**Published:** 2017-04-05

**Authors:** Davide Degli Esposti, Athena Sklias, Sheila C. Lima, Stéphanie Beghelli-de la Forest Divonne, Vincent Cahais, Nora Fernandez-Jimenez, Marie-Pierre Cros, Szilvia Ecsedi, Cyrille Cuenin, Liacine Bouaoun, Graham Byrnes, Rosita Accardi, Anne Sudaka, Valérie Giordanengo, Hector Hernandez-Vargas, Luis Felipe Ribeiro Pinto, Ellen Van Obberghen-Schilling, Zdenko Herceg

**Affiliations:** 1 Epigenetics Group, International Agency for Research on Cancer (IARC), 150 Cours Albert Thomas, 69008 Lyon, France; 2 Instituto Nacional de Cancer, Rio de Janeiro, Brazil; 3 Université Côte d’Azur, CNRS, Inserm, Institut de Biologie Valrose (iBV), 06100 Nice, France; 4 Centre Antoine Lacassagne, Nice, 06189 France; 5 Environment Section, International Agency for Research on Cancer (IARC), 150 Cours Albert Thomas, 69008 Lyon, France; 6 Infection Cancer Biology Group, International Agency for Research on Cancer (IARC), 150 Cours Albert Thomas, 69008 Lyon, France; 7 Université Côte d’Azur, Laboratoire de Virologie, CHU Nice-Archet, 06202 Nice, France; 8 MTA-DE Public Health Research Group, University of Debrecen, Debrecen, Hungary

**Keywords:** Head and neck squamous cell carcinomas, HPV, Differentially methylated regions, CpG shores, Predictive models

## Abstract

**Background:**

Head and neck squamous cell carcinomas (HNSCCs) represent a heterogeneous group of cancers for which human papilloma virus (HPV) infection is an emerging risk factor. Previous studies showed promoter hypermethylation in HPV(+) oropharyngeal cancers, but only few consistent target genes have been so far described, and the evidence of a functional impact on gene expression is still limited.

**Methods:**

We performed global and stratified pooled analyses of epigenome-wide data in HNSCCs based on the Illumina HumanMethylation450 bead-array data in order to identify tissue-specific components and common viral epigenetic targets in HPV-associated tumours.

**Results:**

We identified novel differentially methylated CpGs and regions associated with viral infection that are independent of the anatomic site. In particular, most hypomethylated regions were characterized by a marked loss of CpG island boundaries, which showed significant correlations with expression of neighbouring genes. Moreover, a subset of only five CpGs in a few hypomethylated regions predicted HPV status with a high level of specificity in different cohorts. Finally, this signature was a better predictor of survival compared with HPV status determined by viral gene expression by RNA sequencing in The Cancer Genome Atlas cohort.

**Conclusions:**

We identified a novel epigenetic signature of HPV infection in HNSCCs which is independent of the anatomic site, is functionally correlated with gene expression and may be leveraged for improved stratification of prognosis in HNSCCs.

**Electronic supplementary material:**

The online version of this article (doi:10.1186/s13073-017-0419-z) contains supplementary material, which is available to authorized users.

## Background

Human papilloma virus (HPV)(+)-associated head and neck squamous cell carcinomas (HNSCCs) have been recently shown to present characteristic mutations in various cancer-associated genes, including mutations and focal amplifications of the oncogene *PIK3CA* and cell cycle gene *E2F1*, and truncating mutations in *TRAF3* [1]. Recently, several studies have used gene-specific and genome-wide approaches to examine epigenetic changes such as DNA methylation in HNSCCs [1–9]. These studies identified hypermethylated CpGs in the promoter of a few genes associated with HPV(+) status. However, the intrinsic heterogeneity of the disease and the relatively limited sample size of individual studies tend to limit the power of identifying the impact of HPV on DNA methylation in HNSCCs. To address these limitations and to identify common HPV epigenetic targets in HNSCCs across different anatomical sites with greater power, we performed a pooled analysis of epigenome-wide data in HNSCCs based on the Illumina HumanMethylation450 bead-array data. Finally, we tested whether a unique HPV-induced DNA methylation signature might be found in HNSCCs and might be of clinical relevance in addition to other current diagnostic and prognostic markers, such as p16 immunohistochemical staining or E6/E7 viral mRNA testing.

## Methods

### Cohorts

Thirty-eight HNSCCs from a French cohort (FITMANET, CAL/CHU Tumour bank) included 9 HPV(+) and 29 HPV(–) fresh frozen biopsies. For this cohort, Illumina Infinium HumanMethylation 450 K (HM450K) was performed on a subset of 6 HPV(+) and 6 HPV(–) cases. The remaining cases were used for the validation of the predictive signature by pyrosequencing.

**Fig. 1:**
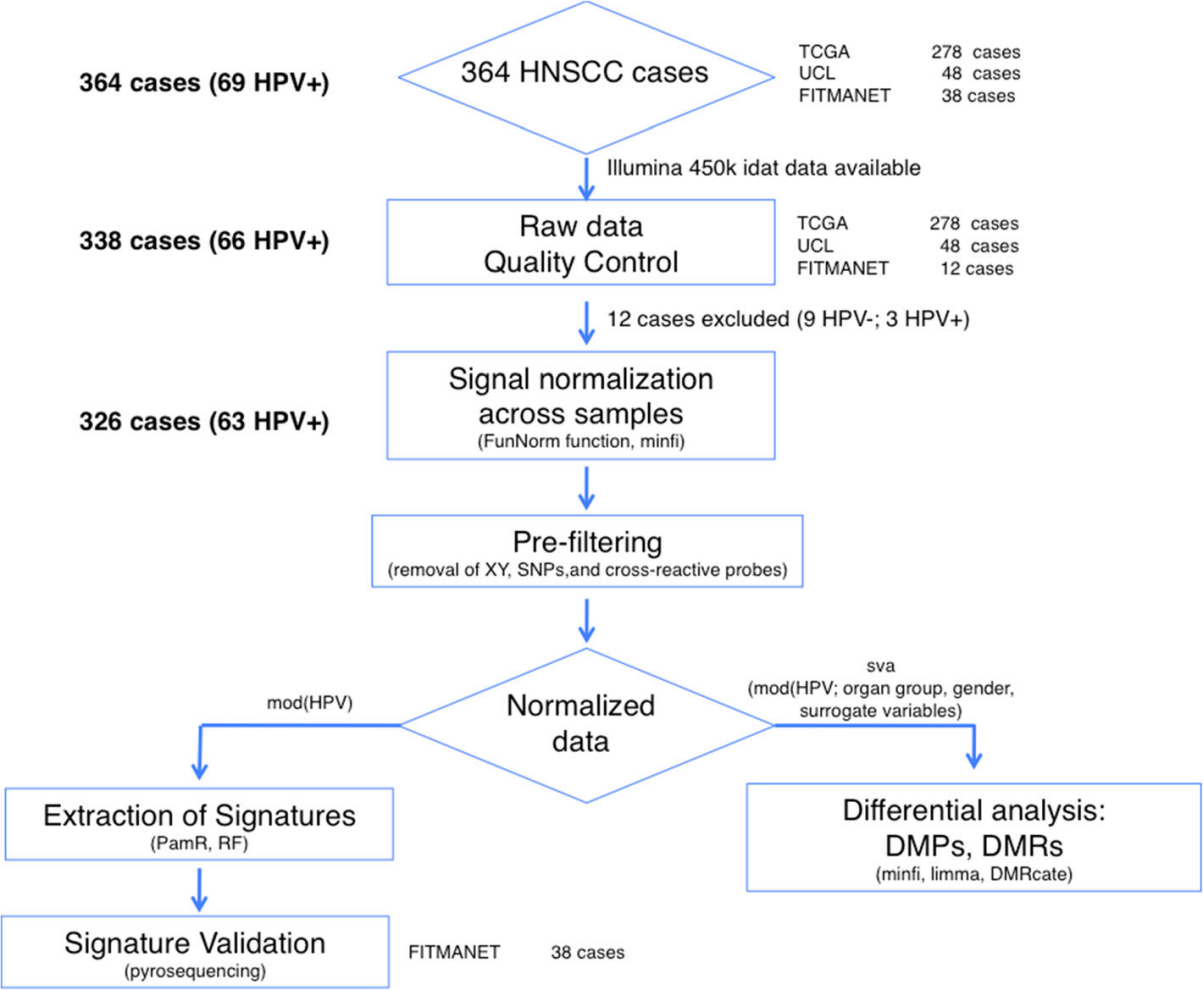
Flow chart illustrating the overall design of the study

The second cohort comprised 278 HNSCC cases from The Cancer Genome Atlas (TCGA) cohort, 36 HPV(+) and 243 HPV(–) [1]. We excluded one HPV(–) case from the original dataset since it was the only tumour localized in the lip. For each case, HM450K data were available and retrieved from the TCGA portal as described in the following sections. The third cohort consisted of 48 oropharyngeal SSCs, 24 HPV(+) and 24 HPV(–) from a study conducted at University College London (UCL) Cancer Institute [2]. Forty-two cases were microdissected from formalin-fixed paraffin-embedded (FFPE) tissues, and 6 were fresh frozen biopsies. Figure 1 shows the overall design of the study.

For the FITMANET and TCGA cohorts, anatomic locations of the tumours were detailed. In particular, 10 anatomic locations were considered. For the UCL cohort, all cases came from the oropharynx, and details on sublocalization were not available. Thus, they were all considered as originating from the oropharynx. In our analysis, we grouped the 10 organs into three distinct anatomic sites, namely oral cavity (alveolar ridge, buccal mucosa, floor of mouth, hard palate oral cavity, oral tongue), oropharynx (base of the tongue, tonsils, oropharynx) and neck (hypopharynx and larynx).

### Determination of HPV status

In the FITMANET cohort, the HPV status was determined by NucliSENS EasyQ HPV, an mRNA-based assay which directly detects active HPV oncogenes (E6/E7 mRNA) in tumour RNA. In the TCGA cohort, HPV status was defined by an empiric definition of more than 1000 mapped RNA-sequencing reads to viral genes E6 and E7 [1]. In the UCL cohort, HPV status was determined by CDKN2A (p16) immunostaining and concomitant E6 qPCR on the DNA extracted from each sample [2].

### Pooled analysis of DNA methylome data

The raw data (.idat files) were downloaded from the TCGA cohort and the UCL cohort. The idat files of the FITMANET cohort were generated by us. All idat files were obtained using the HM450K BeadChip assay, which interrogates more than 480,000 CpG sites. We collected all the idat files from the three cohorts for a total of 338 cases, of which 66 were HPV(+). Then we pooled, preprocessed and normalized the data using the R packages minfi (prefiltering, quality control and normalization using the funnorm function) [10, 11] (Fig. 1). Inferred beta values were used to predict gender as a first quality-control step using the minfi function getSex. All samples were correctly predicted. A prefiltering step was performed in order to keep only samples which were technically successful. We excluded 12 samples (3 HPV(+) and 9 HPV(–)) for which unmethylated and methylated median log_2_ intensities were less than 10.5 (default parameters). Among the excluded samples, 11 cases were from the UCL cohort, whereas one case was from the FITMANET cohort.

A principal component analysis (PCA) was used to determine whether technical aspects of our pooled design (cohort, batch ID, tissue conservation microarray slide or position) might influence variation within the dataset (Additional file 1). As expected, many technical aspects accounted for a significant portion of the variation in the first two principal components, in particular, sentrix ID, cohort, tissue conservation and batch ID. To correct this, we used surrogate variable analysis (sva package) [12], including HPV status, anatomic site and gender in the full model and anatomic site and gender in the null model, and we verified the effect of sva on our data, again using PCA (Additional file 1). For the stratified analysis, we proceeded similarly, but omitted the adjustment for anatomic site. Most of the technical variability was actually corrected, with HPV status becoming the most significant in the first principal component (Additional file 1).

Multidimensional scaling (MDS) plots were generated using the mdsPlot function of the R package minfi. MDS plots were used as an exploratory analysis in order to assess whether distances between samples were associated with specific covariates (HPV status, smoking status, alcohol consumption, organ site). Missing data, in particular for smoking and alcohol consumption, were excluded from the analysis.

### DNA methylome measurement

DNA from the FITMANET cohort was extracted using the QIAamp DNA mini kit (Qiagen) and quantified with Nanodrop (Thermo Scientific). Bisulfite conversion and DNA methylome profiling were carried out as previously described [13, 14].

### Identification of differentially methylated positions (DMPs) and differentially methylated regions (DMRs)

Identification of DMPs was performed as described previously [15, 16]. HPV status associated CpGs were selected based on an adjusted *p* value (false discovery rate, FDR) less than 0.05 and using a differential methylation threshold of 20%. While this threshold may lead to an underestimation of HPV-related methylation changes, we estimated this to be a better conservative approach that may avoid the identification of (or reduce the likelihood of identifying) false positive hits due to undetectable and/or not adjustable batch effects by the surrogate variable analysis applied in the study. Identification of DMRs was performed using the DMRcate R package [17]. Statistically significant DMRs (FDR <0.05) with at least three consecutive CpGs included in a bookend of 1000 nucleotides were retained for further analysis.

### Transcription factor motif enrichment

We analysed the top 500 hypomethylated and hypermethylated regions to identify potential enrichment of any transcription factor motifs in these regions, using HOMER [18].

### Co-methylation plots

Co-methylation analysis and relative plots were generated using coMET, an R package and online tool for visualization of epigenome-wide association study (EWAS) results in a genomic region of interest [19].

### DNA methylation-gene expression correlation analysis

We performed a correlation analysis between DNA methylation and the closest gene expression changes for the DMRs with Δβ_max_ >20% for the TCGA cohort. The gene expression data (expressed in RSEM) were retrieved using the data available in the cBioPortal for Cancer Genomics (www.cbioportal.org) for each gene. In order to calculate the fold change between HPV(+) and HPV(–) cases, we calculated the average expression levels per HPV status for each gene. We used a starburst plot to show all the genes presenting DMRs with Δβ_max_ >20%, using a similar approach as described in Noushmehr et al. (2010) [20].

### Extraction of HPV predictive signatures

Normalized *M* values were analysed with two class prediction algorithms. We used the prediction analysis of microarrays (PAM) algorithm [21] and a random forest (RF) algorithm [22] to evaluate whether CpG methylations were associated with HPV status. For each algorithm, we used HPV status (positive or negative) as the class for prediction. We randomly split the pooled datasets into a training and a test set before running the analysis (training:test set ratio 1:1). We performed five cross-validations with five distinct training sets and testing sets randomly selected from all the cases. The predictive performance of both algorithms was assessed by the area under the receiver operating characteristic (ROC) curve (AUC). We used the average methylation levels of the most recurrent five CpGs across the five cross-validations to create an average methylation index (AMI), and we set a β-value cut-off of 0.75 (corresponding to 75% of methylation in the pyrosequencing results): samples with AMI lower than 0.75 were classified as HPV(+).

### Pyrosequencing analysis

Bisulfite-treated DNA was amplified using primers targeting CpGs in the B3GALT6-SDF4 locus (cg13924635, cg22220310, cg14477263), HLTF-HLTF-AS1 locus (cg07275648) and SYCP2-FAM127B locus (cg21950459), identified by the two class predictors. DNA methylation levels at single CpG sites were determined by pyrosequencing using PyroMark Q96 ID (Qiagen).

### Survival analysis

We performed a Kaplan-Meier survival analysis in the TCGA cohort using the survival data reported in Lawrence et al. [1]. We included all the patients for which days to death or days to last follow-up were available, thus including 277 patients. We used either the AMI we built on the five-CpG signature or the HPV status as determined by viral gene expression using RNA-sequencing data. Data were analysed using the R package survival.

## Results

### HPV(+) HNSSCs show a tissue-independent DNA methylation signature across anatomic sites

**Fig. 2:**
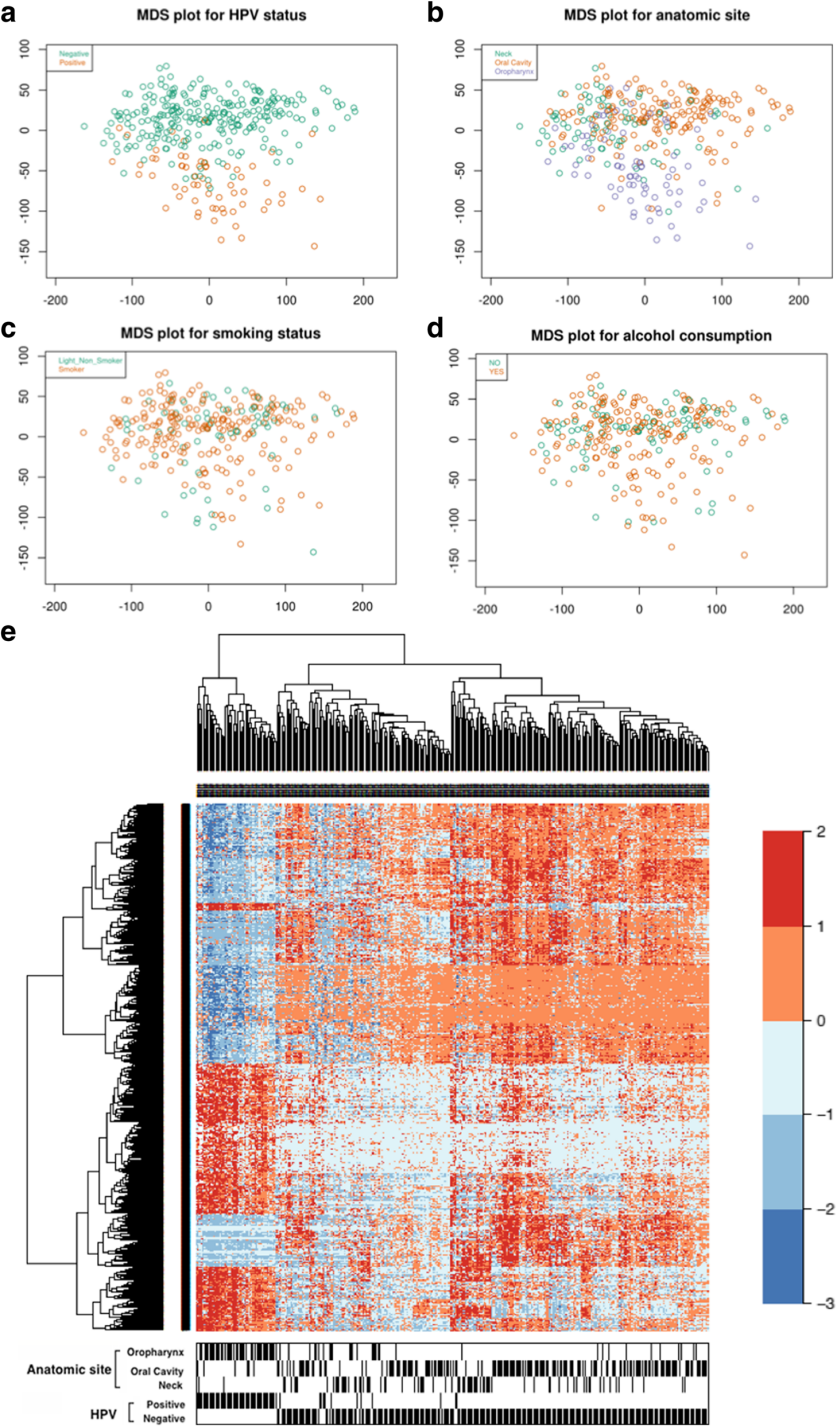
HPV infection leaves clear DNA methylation signature in HNSCCs. MDS plots showing sample clustering grouped by different variables: **a** HPV status, **b** organ site, **c** smoking status, **d** alcohol consumption. **e** Heatmap showing the 2410 DMPs associated with HPV status (FDR <0.05, Δβ >20%)

To address the impact of HPV infection on the DNA methylome in HNSCC with greater power, we collated two major studies with raw DNA methylome data [1, 2] and performed additional methylome analyses of 6 HPV(+) and 6 HPV(–) HNSCC samples from a smaller independent French cohort (FITMANET). Thus, in the analysis we included the methylome data from different geographical regions with a total of 338 cases, among which 66 were HPV(+) (Table 1, Fig. 1). After applying stringent quality-control filters (see Methods section), we analysed the methylome of 326 cases, of which 63 were HPV(+). An initial multidimensional scaling (MDS) analysis, using normalized logit-transformed methylation values for the most variable probes (4500 probes) to calculate their Euclidean distance, showed that cancer samples from the three datasets clustered clearly in two separate groups based on their HPV status (Fig. 2a, Additional file 2: Figure S1A–C). Importantly, these clusters did not associate with anatomic site, smoking habits and alcohol consumption (Fig. 2b–d).

**Table 1:** Patient characteristics of the head and neck cancers for which DNA methylome data were analysed

Patient characteristics	HPV positive No. cases (%)^a^	HPV negative No. cases (%)^a^
Pooled cases	66 (100)	272 (100)
TCGA	36 (54.5)	242 (89.0)
UCL	24 (21 FFPE) (36.4)	24 (21 FFPE (8.8)
FITMANET	6 (9.1)	6 (2.2)
Male	55 (83.3)	195 (71.7)
Female	11 (17.7)	77 (28.3)
Median age (range)	59 (35–86)	62 (19–90)
Tobacco smoking		
Never/light smokers	17 (25.7)	54 (19.8)
Smokers	25 (37.9)	218 (80.0)
Not available	24 (36.4)	25 (9.2)
Alcohol consumption		
No	9 (13.6)	83 (30.5)
Yes	32 (48.5)	159 (58.5)
Not available	25 (37.9)	30 (11.0)
Tumour stage		
T1	8 (12.1)	13 (4.8)
T2	19 (28.8)	49 (18.0)
T3	9 (13.6)	64 (23.5)
T4 (A + B)	28 (42.4)	118 (43.4)
Not available	2 (3.1)	28 (10.3)
Tumour site		
Oral cavity	12 (18.2)^b^	162 (59.5)
Oropharynx	51 (77.3)^c^	35 (12.9)
Neck (hypopharynx and larynx)	3 (4.5)^d^	75 (27.6)

^a^Percentage calculated using the total number of cases in the group of reference

^b^HPV16: 8 cases, HPV33: 4 cases

^c^HPV16: 48 cases, HPV33: 3 cases (1 co-infection with HPV16), HPV35: 1 case

^d^HPV16: 3 cases (2 cases in hypopharynx, 1 case in larynx)

We identified differentially methylated positions (DMPs, FDR <0.05 and differential methylation, Δβ >20%) in the whole dataset, after gender and anatomic site adjustment (see Methods section). Unsupervised clustering showed that 2410 DMPs (1023 hypermethylated CpGs, mapping to 510 genes; 1387 hypomethylated CpGs, mapping to 756 genes, for a total of 1266 genes) comprised one distinct cluster that includes the majority of HPV(+) cases (53/63, 84%) (Fig. 2e). To rule out that these differences are due to the different HPV prevalence in the different organ sites (59%, 7% or 4% in oropharynx, oral cavity and hypopharynx-larynx, respectively), we tested whether the 2410 DMPs found in the whole datasets were able to correctly identify HPV(+) cases in the different organ sites. As shown in Additional file 2: Figure S2A–C, most HPV(+) cases (53/63) clustered together in the different organ sites, indicating that these DMPs are associated to the HPV status independently of the anatomic site.

Then, we stratified the tumours for anatomic site (adjusting for gender) and found organ-specific patterns of differential methylation associated with HPV status, without evident cohort effects (Additional file 2: Figure S1D–F). However, in this case the different prevalence of HPV in the different organ groups and the low number of cases may hamper the significance of an HPV signature specific for each different tissue or organ group. Thus, the tissue-specific epigenetic impact of the virus remains to be addressed.

We compared the list of the 1266 genes for which we found HPV(+)-associated DMPs with other DNA methylation changes of HPV(+) HNSCC, obtained with different genome-wide platforms (Illumina Golden Gate or Illumina HumanMethylation27 bead-array, MCIp-Agilent 244 K). While a formal comparison with the published signatures was not possible due to the lack of overlap at single CpG sites across the platforms, we comprehensively included all the genes mentioned in each paper (Additional file 3). This comparison revealed that our HPV(+) signature included many of the hypermethylated genes previously described [2, 3, 5, 7–9] (Additional file 3). Our analysis also identified hypermethylation of new genes associated with HPV status, including two other cadherin-associated genes (*CDH18* and *CTNND2*) for which methylation levels were not previously described as deregulated in HPV(+) tumours. A systematic ad hoc search for biological functions indicated that hypermethylated genes shared by all anatomic sites are mainly involved in neuronal differentiation, calcium signalling and encoding of DNA-binding zinc finger proteins, the latter previously observed in another study [3] (Additional file 3).

Interestingly, our analysis also revealed that 60% of differentially methylated genes (756 out of 1266) associated with HPV(+) HNSCCs were hypomethylated. The main biological functions associated to the hypomethylated genes common to all anatomic sites were apoptosis, cell cycle regulation and non-coding RNAs (ncRNAs), of which two have been described as deregulated in leukemias (*DLEU1* and *mir9*-*3*) (Additional file 3).

### Hypomethylated regions in CpG shores are associated with HPV infection and activated gene expression

**Fig. 3:**
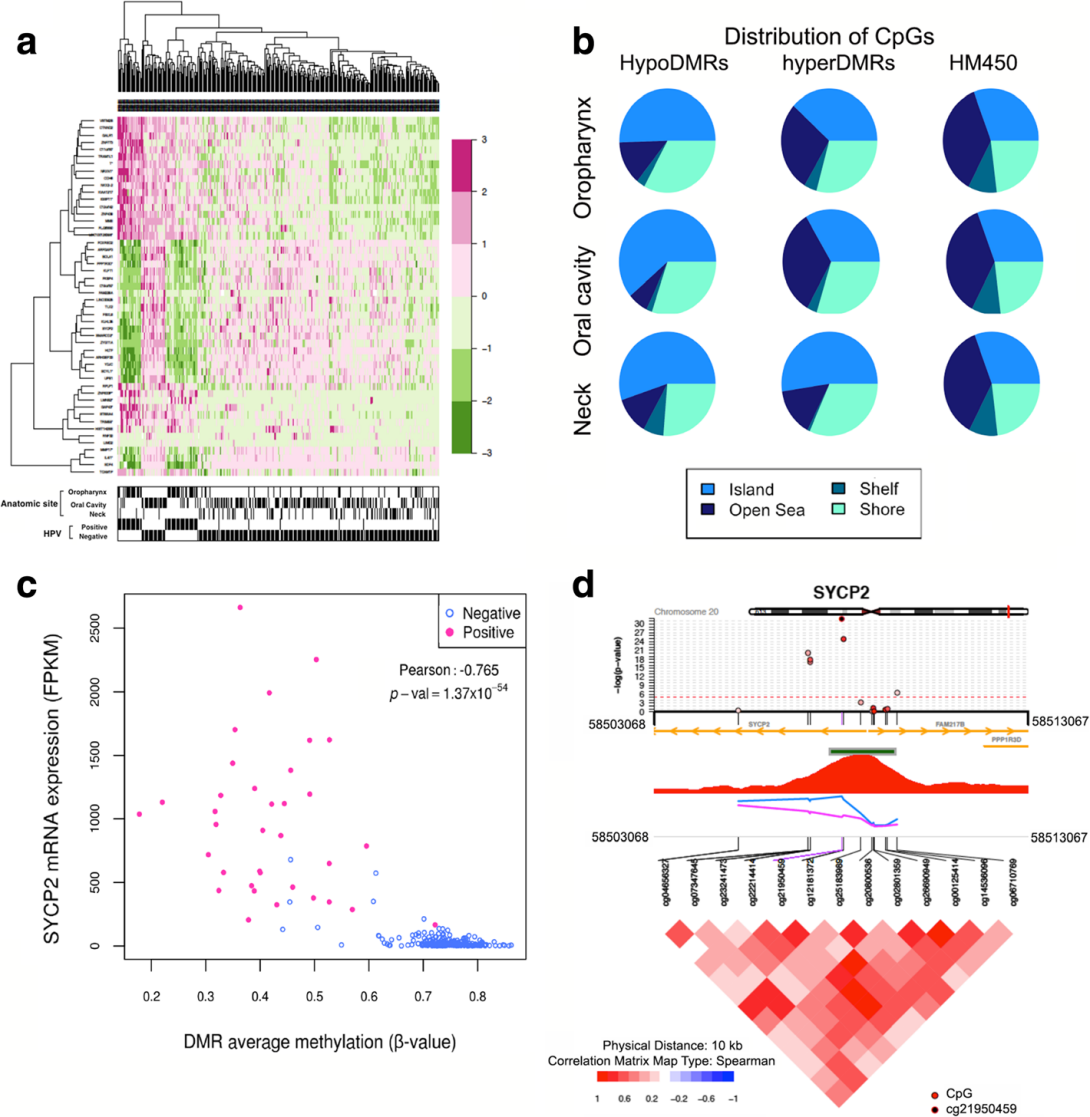
Hypomethylated DMRs associated with HPV infection show CpG island shore loss of boundary and functional correlation with gene expression. **a** Heatmap showing the top 50 DMRs associated with HPV status (FDR <0.05). **b** CpG context of the identified DMRs in the different anatomic sites compared with their distribution in the Illumina HumanMethylation 450 K array (HM450). **c** Correlation between CpG methylation at the SYCP2 DMR and relative gene expression in the HNSCC cases of the TCGA cohort. HPV(+) cases are indicated by *full pink dots*, HPV(–) cases by *empty blue dots*. **d** Co-methylation plots showing the CpGs in SYCP2 DMR ranked by *p* value and visualized based on their chromosomal coordinates, relative position to CpG island (*green bar*) and CpG content (*red peak*). The average methylation values in the DMR in HPV(+) (*pink*) or HPV(–) (*blue*) cases are shown. The correlation plot shows Spearman correlation values among the CpGs in the region

Since alterations in DNA methylation can affect multiple neighbouring CpG sites, we applied dimension reduction to identify differentially methylated regions (DMRs, at least three CpGs in a 1-kb region), using the DMRcate package (see Methods for details) in the whole dataset (Additional files 4 and 5) and in each stratified organ group (Additional files 6, 7 and 8). Globally, we identified 4371 hypermethylated DMRs and 2044 hypomethylated DMRs (FDR <0.05). However, using more stringent criteria (increasing the differential methylation to Δβ_max_ >20% or Δβ_max_ >30%), we found an increased proportion (from 32% to 65% or 95%, respectively) of hypomethylated regions associated with HPV(+) status (Table 2). Interestingly, we found that average methylation levels of the top 25 hypomethylated and top 25 hypermethylated DMRs (Additional files 4, 5, 6, 7 and 8) were able to separately cluster HPV(+) from HPV(–) HNSCCs for each anatomic site (57/63) (Fig. 3a, Additional file 2: Figure S2D–F). Globally, both hyper- and hypomethylated regions presented an enrichment not only in CpG islands, as previously described, but also in shores (Fig. 3b), thus revealing new genomic regions targeted by DNA methylation changes in HPV(+) HNSCCs.

**Table 2:** Number of differentially methylated regions (DMRs) identified by DMRcate (minimum three CpGs in a 1-kb window) based on the maximum differential methylation in the region

Δβ threshold	Hypomethylated DMRs	Hypermethylated DMRs
No threshold	2044 (32%)	4372 (78%)
Δβ_max_ >5%	1716 (32%)	3788 (78%)
Δβ_max_ >10%	797 (33%)	1592 (67%)
Δβ_max_ >20%	112 (65%)	61 (35%)
Δβ_max_ >30%	20 (95%)	1 (5%)

**Fig. 4:**
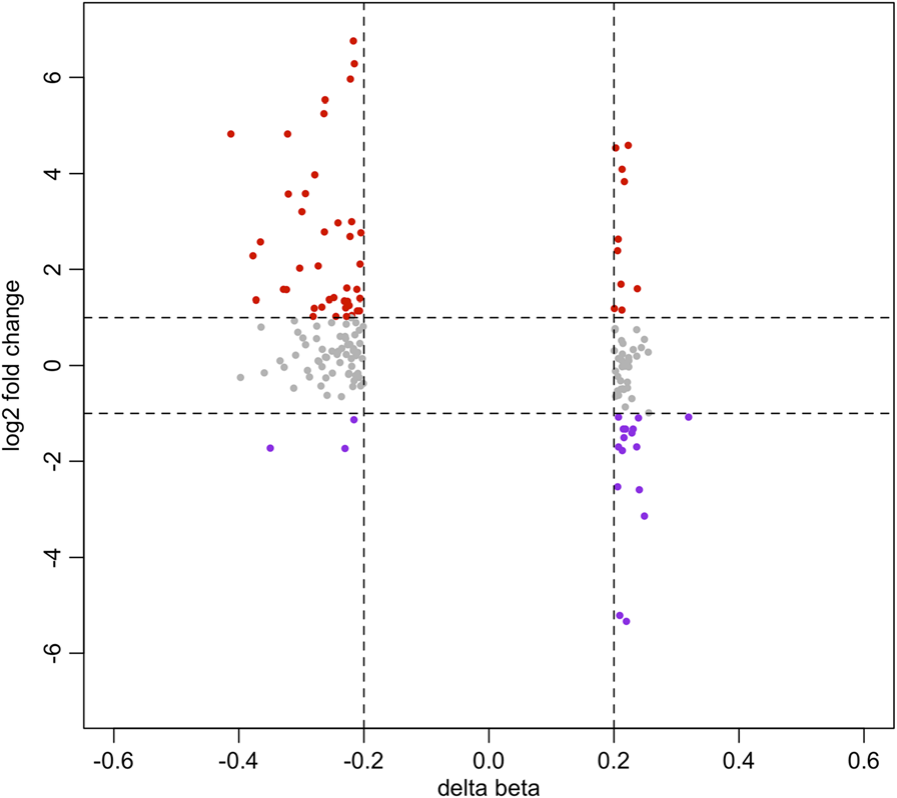
DNA methylation changes associated with gene expression changes in the TCGA cohort. Starburst plot showing correlations between gene expression changes and DNA methylation changes in DMRs with Δβ >20%. *Red dots* indicate up-regulated genes in HPV(+) cases with a minimum logFC >1. *Purple dots* indicate down-regulated genes with a minimum logFC < –1. *Grey dots* indicate genes with expression changes –1 < logFC < 1 and changes in methylation with Δβ >20%

In order to investigate potential functional effects of DNA hypomethylation in these regions, we first plotted gene expression log_2_ fold changes (log_2_FC) and DNA methylation changes (Δβ_max_ >20%) of the genes belonging to the DMRs found with a Δβ_max_ >20%. Gene expression data were available for 165 genes (105 hypomethylated and 60 hypermethylated) for the TCGA cohort and were extracted from the cBioPortal. We found that 49 out of 105 genes located in hypo-DMRs showed an increased gene expression (log_2_FC >2), while 15 out of 60 genes located in hyper-DMRs showed decreased gene expression (log_2_FC <2) (*p* value < 0.006, χ^2^ test) (Fig. 4). We then systematically analysed the 50 most differentially methylated regions (Stouffer FDR <0.05; ranked by Δβ_max_). Similarly, we found a significant correlation (Pearson, *p* value < 0.05) between average DNA methylation levels and gene expression in 52% (13 out of 25 cases) of the genes located in hypo-DMRs and in 4% (1 out of 25 cases, *ZNF733*) of the genes located in hyper-DMRs (*p* value < 0.001, χ^2^ test) (TCGA dataset) (Additional file 9).

As in previous studies, we observed an inverse correlation between DNA methylation and gene expression (Fig. 3c, Fig. 4, Additional file 9). Unexpectedly, these results suggest that hypermethylated DMRs are less likely to affect gene expression than hypomethylated DMRs in this context (Additional file 9). The number of analysed genes whose expression was affected by DNA methylation changes in the DMRs was too low to get meaningful results from pathway analyses. However, we found three hypomethylated genes which were overexpressed in HPV(+) HNSCCs, all of which are involved in the extracellular matrix organization (*NCAN*, *NRXN1*, *COL19A1*). Other hypomethylated genes whose expression was increased in HPV(+) cases were involved in structural maintenance of chromosomes during mitosis/meiosis (*SYCP2*, *RPA2*, *SMC1B*). The package CoMet plot [19] was next used to visualize regional epigenome-wide co-methylation associations and to expand the analysis to a 10-kb region around each DMR. We found that methylation patterns at the single CpG level were significantly and positively correlated when located in close proximity to the DMRs (Fig. 3d, Additional file 9). Moreover, most DMRs which showed a correlation with gene expression presented a marked loss of CpG island boundaries (Fig. 3d, Additional file 9), a feature previously described in other cancer types and associated with cancer development [23, 24]. A similar trend was observed for genes besides the top 25 DMRs systematically analysed (such as *MEIS1*, *STAT5A*), suggesting that the functional impact of DNA hypomethylation in the CpG shores on gene expression may be generalized (Additional file 9).

Interestingly, our search for transcription factor binding sites (TFBSs) across DMRs showed an enrichment of TFBS motifs related to *c*-*MYC* in hypomethylated DMRs. Actually, *c*-*MYC* is typically amplified in HPV16 cervical squamous cell carcinomas [25, 26], and its binding to the consensus DNA element is reported to be methylation-sensitive, in particular at CpG islands and nearby regions, supporting our finding of an interaction at hypomethylated regions at CpG island shores [27, 28].

### HPV(+) HNSSCs showed an organ-site-independent DNA methylation signature of clinical relevance

**Fig. 5:**
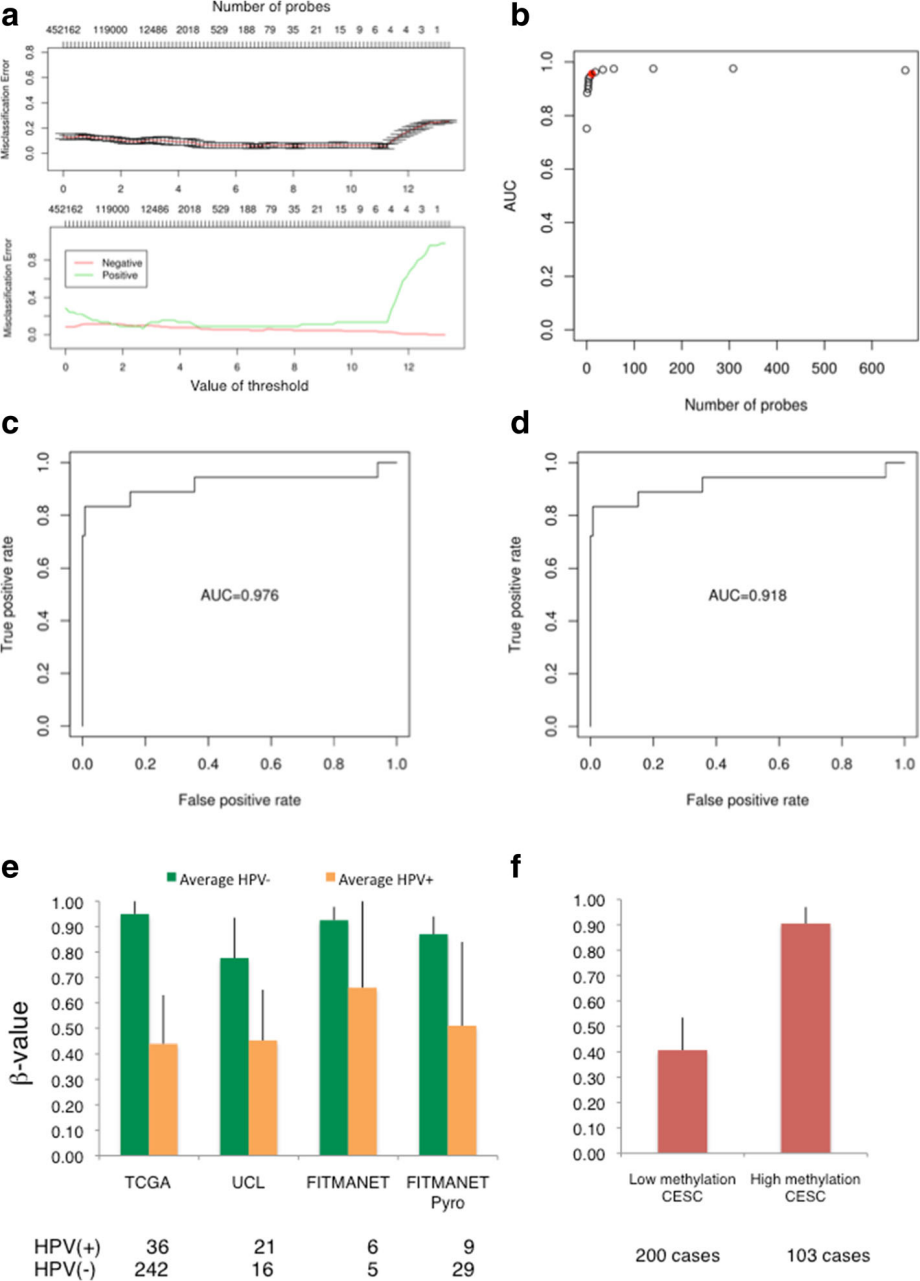
DNA methylation predictive signature of HPV status in HNSCCs. **a** Overall and class-specific misclassification errors based on the number of CpGs selected to predict HPV status. **b** AUC values from PAM algorithm according to the number of probes selected to predict the HPV status. The *red dot* indicates the AUC for 10-CpG signatures. Similar results were obtained using RF algorithm (data not shown). **c** Receiver operating characteristic (*ROC*) curves (AUC) using the training set data. **d** ROC curves (AUC) using the test set data. **e** Average methylation index (*AMI*) of the 5-CpG signature across the different datasets (TCGA cohort, UCL cohort, FITMANET cohort 450 K, FITMANET validation cohort). **f** AMI of the 5-CpG signature in cervical carcinomas from TCGA. Similar to HNSCCs signature, high methylation is considered when the AMI is higher than 0.75

To test whether HPV-associated methylation changes may be predictive of HPV status, we next analysed the normalized data using a prediction analysis of microarrays (PAM) algorithm and a random forest (RF) algorithm (see Methods section), performing five cross-validations for each method. ROC curve (AUC) analysis showed that methylation values of as low as 10 CpGs (Fig. 5a, b) were able to give AUC >0.95 in most of the training and the test sets (Fig. 5c, d). Predictive signatures of 10 CpGs for each cross-validation resulted in an average sensitivity of 89% (87–94%) and an average specificity of 96% (95–97%) in the training test. In the test set, the average sensitivity was 89% (83–94%) and the average specificity was 95% (range 94–97%). The recurrent CpGs across the five cross-validated signatures of 10 CpGs were localized in the B3GALT6-SDF4 locus (3 CpGs), in the SYCP2-FAM217B locus (1 CpG), the HLTF-HLTF-AS1 locus (from 1 to 3 CpGs), *TLX2* gene (from 1 to 2 CpGs), LOC729683 (from 1 to 2 CpGs), *IL4I4* gene (1 CpG) and LINC00925 (1 CpG).

To validate the predicted signatures using alternative quantitative methods, such as pyrosequencing, we selected the five most recurrent CpGs appearing in the 10-CpG signatures of each cross-validation. In particular, we used the three CpGs localized in the B3GALT6-SDF4 locus (cg13924635, cg22220310, cg14477263), in the SYCP2-FAM217B locus (cg21950459) and in the HLTF-HLTF-AS1 locus. We used the average methylation levels of these five CpGs to create an average methylation index (AMI), and we set a β-value cut-off of 0.75 (corresponding to 75% of methylation in the pyrosequencing results) (see  Methods). By applying this approach to the entire French cohort (38 cases, 9 HPV(+)), we obtained a sensitivity of 66.7% and a specificity of 93.1%, further verifying the robustness and predictive power of our HPV-specific methylation signature (Fig. 5e). Finally, we used our 5-CpG signature to stratify 303 cervical squamous cell carcinomas from TCGA and found that 66% of them showed similar levels of methylation compared with HPV(+) HNSCCs (Fig. 5f).

**Fig. 6:**
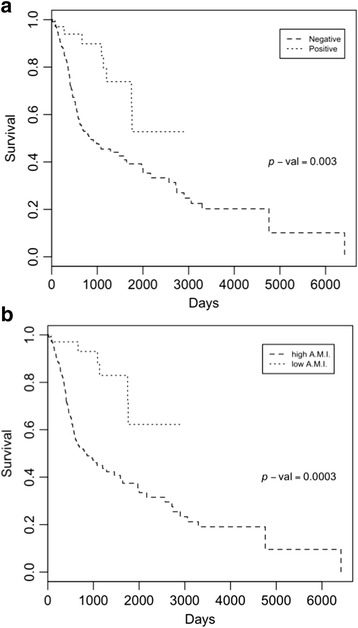
DNA methylation signature predictive of survival in the TCGA cohort. **a** Kaplan-Meier survival curve based on HPV status determined by viral gene expression using RNA-sequencing data. **b** Kaplan-Meier survival curve based on the average methylation index (*AMI*) of the 5-CpG signature. LowAMI corresponds to an AMI less than 0.75, while highAMI corresponds to an AMI higher than 0.75

Finally, we tested whether our 5-CpG signature was a good predictor of survival, since HPV(+) status is associated with better survival than HPV(–) status in HNSCCs. Indeed, when HPV status determined with viral gene expression was used to stratify the patients, patients with HPV(+) showed better survival than patients with HPV(–) after almost 8 years of follow-up (2898 days) (52% vs 27%, *p* value = 0.003, χ^2^ test) (Fig. 6a). Interestingly, for the same follow-up period, we found that patients with a low AMI showed a 62% survival rate, while patients with a high AMI showed a 25% survival rate (*p* value = 0.0003, χ^2^ test) (Fig. 6b). These results indicate that our HPV-associated epigenetic signature may be used as a predictor of survival for HPV(+) HNSSCs.

## Discussion

In this study we present the first pooled analysis of genome-wide DNA methylation data from HM450K in HNSCCs. Compared with the largest cohort (TCGA) for which HM450K data were available, the pooled cohorts have 43% more HPV(+) cases analysed (63 vs 36), leading to a prevalence of 19% of HPV(+) cases. Moreover, many head and neck organs are included, allowing an assessment of HPV methylation signatures in different anatomic sites. Globally, our results show that HPV has a specific and genome-wide effect in shaping the DNA methylome of HNSCCs that is independent of other known major risk factors, such as tobacco smoking and alcohol consumption. Our results are similar to a recent report based on the analysis of the whole TCGA cohort [29]. The authors identified five subclusters of HNSSC based on DNA methylation patterns (528 samples), four HPV(–) clusters and one HPV(+) cluster [29].

Previous works reported aberrant hypermethylation in gene promoters of HPV(+) oropharyngeal cancer cases [2, 3, 9]. Identified target genes differed between the studies: Lleras et al. [3] reported *CDKN2A* and *GALR1* among the most hypermethylated gene promoters; Lechner et al. [2] reported Polycomb repressive complex 2 target genes, mainly cadherin coding genes; while Kostareli et al. [9] reported *ALDH1A2*, *OSR2*, *GATA4*, *GRIA4* and *IRX4* as the most hypermethylated gene promoters. Differences in the various studies may be attributed to the different platforms used (Illumina Methylation 27 K, HM450K, Agilent 244 K), different tissue conservation (frozen or FFPE) and relatively small sample sizes. However, we found a marked overlap with the genes described by Lechner et al. [2], whose dataset was included in our pooled analysis. Moreover, while the different published signatures showed no overlap among the hypermethylated genes, our signature showed partial overlap to each of them (although we cannot exclude that we missed partial overlaps among the published signatures due to partial reporting in the papers). In addition, our analysis identified hypermethylation of new genes associated with HPV status, including two additional cadherin-associated genes (*CDH18* and *CTNND2*) for which methylation levels were not previously described as deregulated in HPV(+) tumours. Interestingly, among the hypermethylated DMRs we systematically analysed in order to look at their potential impact on gene expression, we found a zinc finger factor coding gene (*ZNF733*), a gene family for which promoter hypermethylation and consequent down-regulated expression were previously described in HNSCCs [3].

To date, only few hypomethylated genes have been linked to HPV status [2, 5, 7–9]. Our analyses found 60% of differentially methylated genes were hypomethylated. These results highlight the power of our pooled analysis in identifying novel genes and regions in which methylation levels are influenced by HPV infection. Importantly, we found that hypomethylated DMRs were more likely to affect gene expression than hypermethylated DMRs. This result extends to HNSCC previous observations on hypomethylated blocks in some human solid tumours [23, 24]. Our motif enrichment analysis suggests that *c*-*MYC*-induced immortalization may be facilitated by the hypomethylation of many of its potential target genes. Among the most hypomethylated regions we found to be associated with increased gene expression was that in the CpG island shore of the *SYCP2* gene (Fig. 3). In fact, a recent study describes that *SYCP2* up-regulation predicts early stage HPV(16) oropharyngeal carcinomas [28]. Moreover, we identified a minimal epigenetic signature made of five CpGs encompassing three genetic loci (B3GALT6-SDF4, SYCP2-FAM127B and HTLF-HLTF-AS1) that showed a high specificity (>95%) in identifying HPV(+) cases, independent of the anatomic site. Indeed, one of the CpGs belonging to our 5-CpG predictive signature of HPV status belongs to the SYCP2 DMR, suggesting that its up-regulation is functionally mediated by HPV-associated demethylation at its close CpG island shore. Finally, our signature seems to have potentially important clinical relevance. Notably, we showed that this epigenetic signature was a better predictor of survival compared with HPV status itself (assessed with the highly accurate method of measuring viral gene expression using RNA-sequencing data) in the TCGA cohort (Fig. 6). While further studies are needed to investigate the precise molecular mechanism underlying these findings, one can speculate that this epigenetic signature is able to integrate the different epigenetic alterations induced by the multiple exposures occurred in these patients (notably, heavy smoking, alcohol consumption and HPV infection), resulting in a better discriminator for survival.

## Conclusions

Our results based on genome-wide DNA methylation data from different cohorts show that HPV infection greatly affects DNA methylation in HNSCCs across different anatomic sites. Our analyses are based on a pooled large dataset, and our results expand and complement those already published [30]. As previously described, we observed a higher prevalence of HPV(+) cases in men than in women [31]. A limitation of the analysis regards the lack of complete smoking/alcohol data, as this has limited our ability to assess differences based on smoking. We determined that previously observed hypermethylation in some promoter-associated CpG islands is less likely to correlate with gene expression than the hypomethylated CpG island shores we identified, which showed a strongly significant correlation with the expression of associated genes. As few as five of these methylation changes provided a highly specific predictive signature of HPV status, suggesting that HPV drives some DNA methylation-dependent molecular events, for example, higher nucleosome accessibility to c-Myc transcription factor [32]. Intriguingly, the sensitivity we observed in the validation cohort and the fraction of cervical cancer showing a lowly methylated score (66.7%, 66%, respectively) reflects the HPV16 prevalence in cervical cancer (around 70%) [33], suggesting potential HPV subtype-specific methylation changes, since HPV16 was the most common subtype (89% prevalence) in the HNSCCs analysed in this study. While we do not estimate that our epigenetic signature might replace current diagnostic methods in a short time frame, our signature is a promising predictor of survival for HNSCCs. However, our signature should be tested in larger cohorts of patients with HNSCC, using multivariate statistical models, to improve the power and eventually provide a better marker for establishing more targeted treatments, especially when considering HPV status for de-escalation regimens to avoid long-term toxicity of standard-of-care treatment.

## Additional files

Additional file 1: Model optimization. (PPTX 1128 kb)

Additional file 2: Figure S1. MDS plots and differentially stratified methylation analysis. **Figure S2.** HNSCCs clustering by organ and by top 50 DMRs. (PPTX 1324 kb) (PPTX 1324 kb)

Additional file 3: Published methylation signatures in HNSCCs. Ad hoc pathway analysis of common hyper- and hypomethylated sites. (DOCX 29 kb)

Additional file 4: Hypomethylated regions. (XLSX 372 kb)

Additional file 5: Hypermethylated regions. (XLSX 620 kb)

Additional file 6: Differentially methylated regions in oropharynx. (XLSX 884 kb)

Additional file 7: Differentially methylated regions in oral cavity. (XLSX 171 kb)

Additional file 8: Differentially methylated regions in neck. (XLSX 130 kb)

Additional file 9: Co-methylation analysis. (PPTX 3382 kb)

## Abbreviations

AUC: Area under the curve
DMP: Differentially methylated position
DMR: Differentially methylated region
FDR: False discovery rate
HNSCC: Head and neck squamous cell carcinoma
HPV: Human papilloma virus
PAM: Prediction analysis for microarray
PCA: Principal component analysis
RF: Random forest
TCGA: The Cancer Genome Atlas
TFBS: Transcription factor binding site
UCL: University College London
