# Supplementary material for: Unique DNA methylation signature in HPV-positive head and neck squamous cell carcinomas
Source: Genome Med. 2017 Apr 5;9:33. doi: 10.1186/s13073-017-0419-z (PMC5382363; doi:10.1186/s13073-017-0419-z)
Supplement: Supplementary file 1 — Model optimization. (PPTX 1128 kb) [file 13073_2017_419_MOESM1_ESM.pptx]

## Slide 1
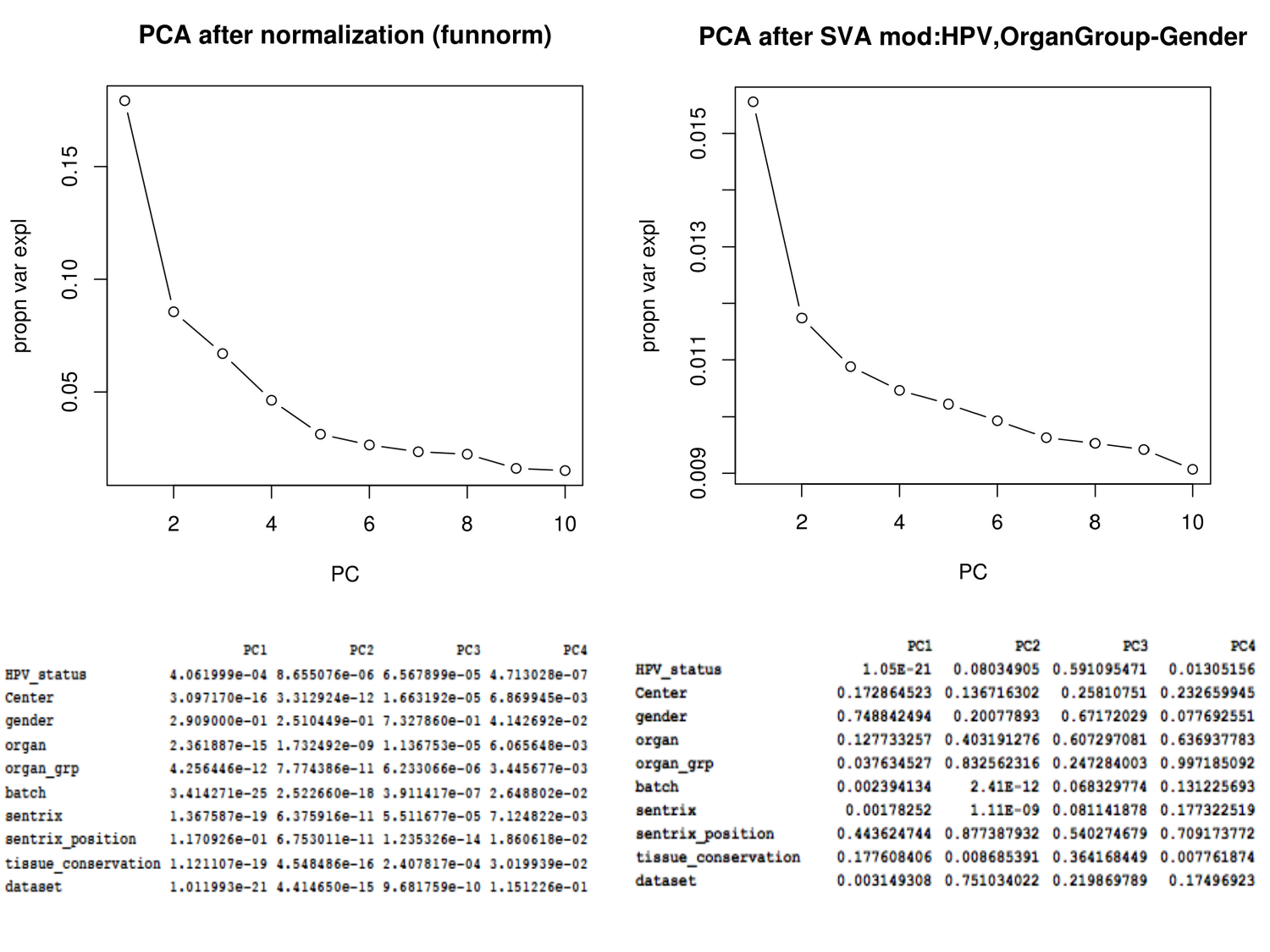

## Slide 2
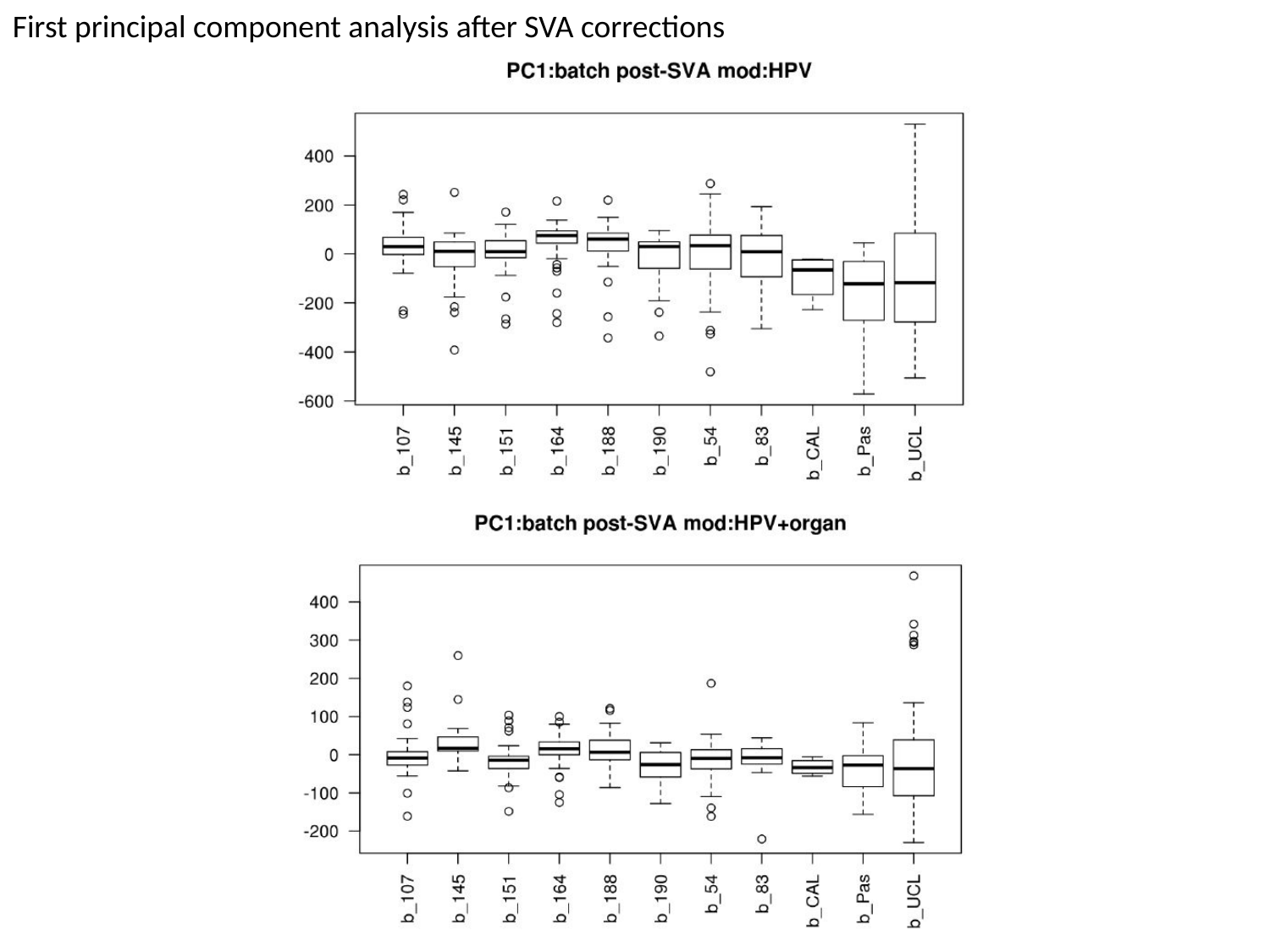

First principal component analysis after SVA corrections

## Slide 3
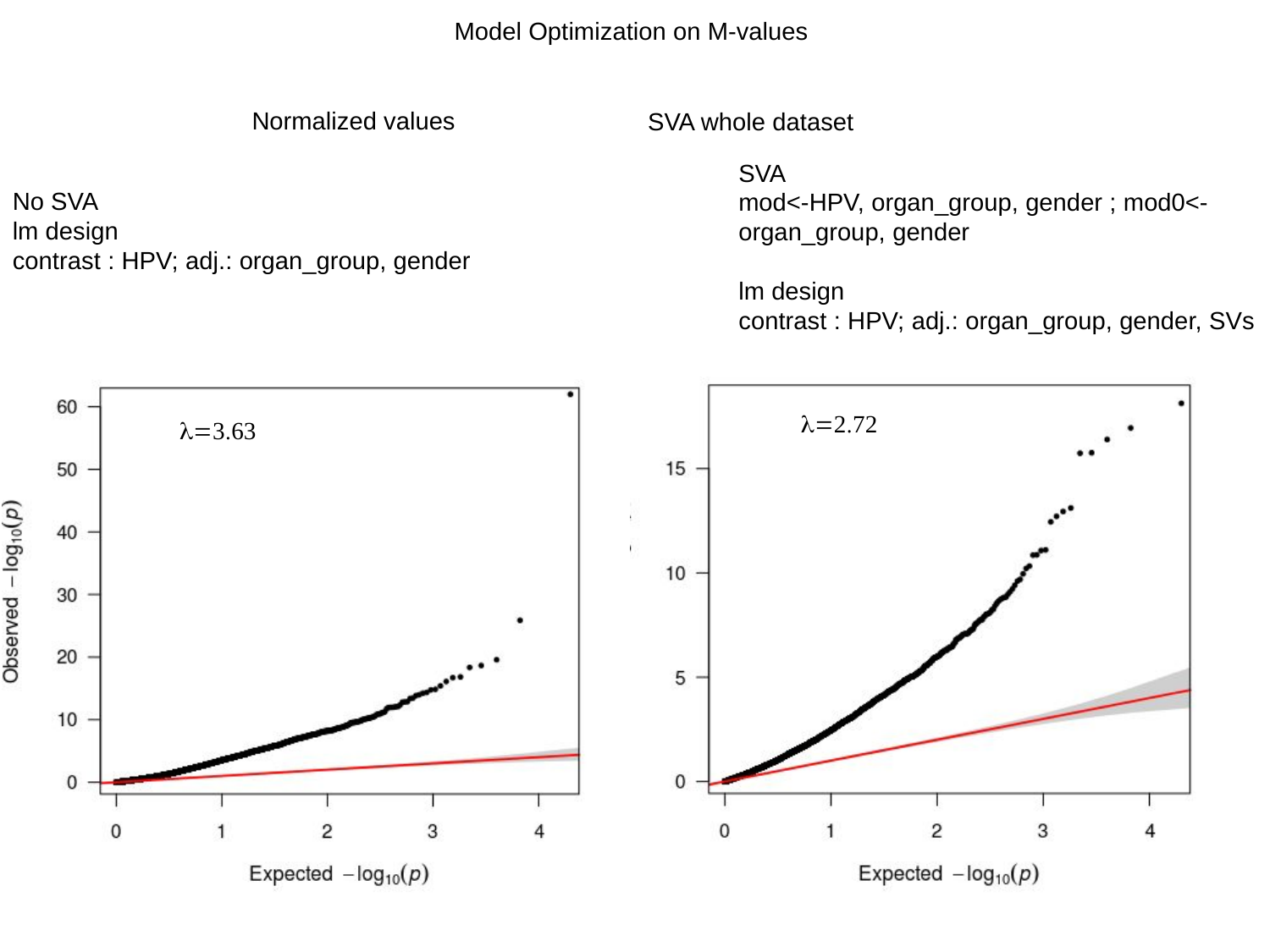

Model Optimization on M-values
Normalized values
SVA whole dataset
SVA
mod<-HPV, organ_group, gender ; mod0<-organ_group, gender
lm design
contrast : HPV; adj.: organ_group, gender, SVs
No SVA
lm design
contrast : HPV; adj.: organ_group, gender
l=2.72
l=3.63

## Slide 4
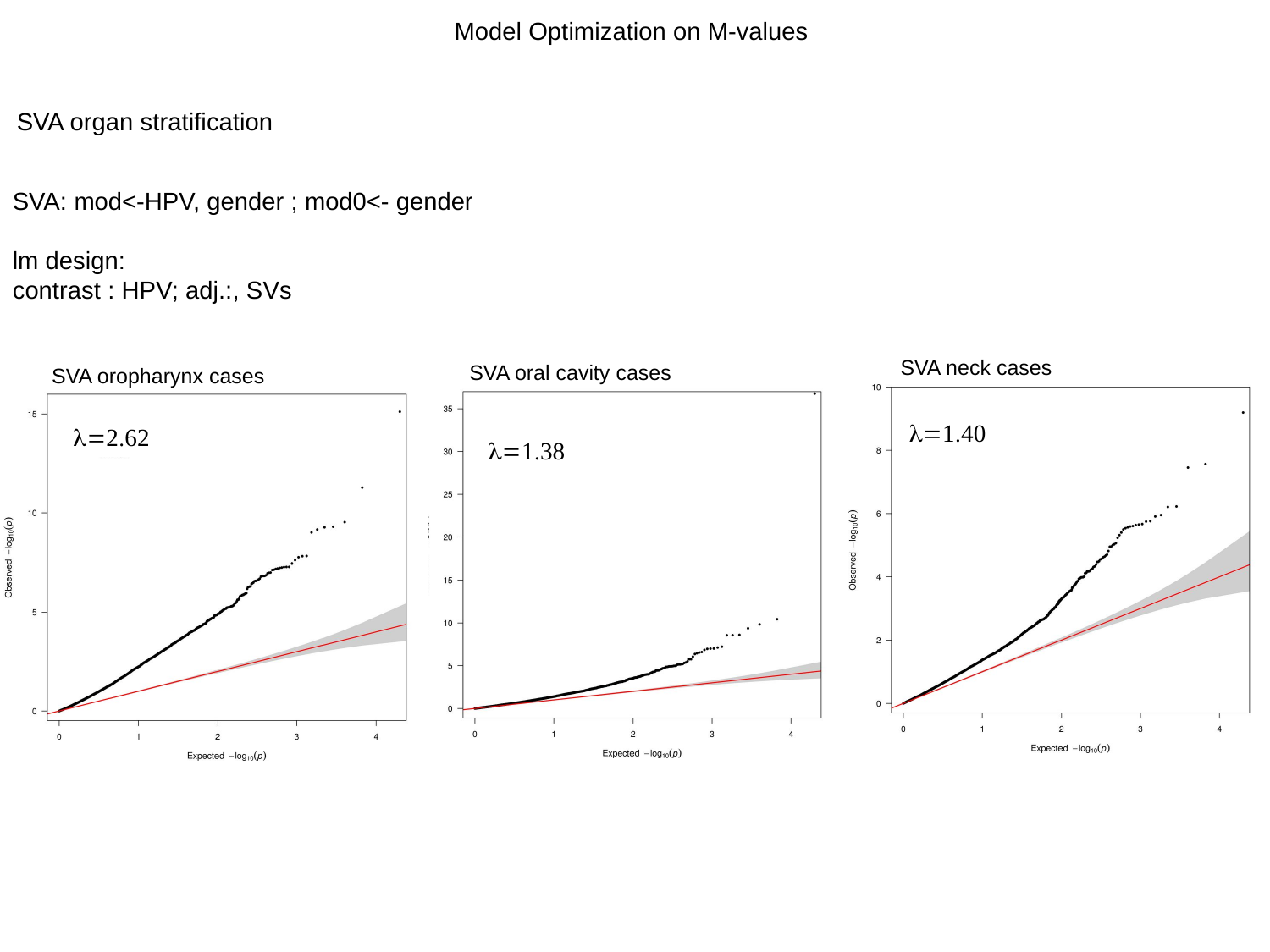

Model Optimization on M-values
SVA organ stratification
SVA: mod<-HPV, gender ; mod0<- gender
lm design:
contrast : HPV; adj.:, SVs
SVA neck cases
SVA oral cavity cases
SVA oropharynx cases
l=1.40
l=2.62
l=1.38
