# Supplementary material for: Unique DNA methylation signature in HPV-positive head and neck squamous cell carcinomas
Source: Genome Med. 2017 Apr 5;9:33. doi: 10.1186/s13073-017-0419-z (PMC5382363; doi:10.1186/s13073-017-0419-z)
Supplement: Supplementary file 2 — MDS plots and differentially stratified methylation analysis. Figure S2. HNSCCs clustering by organ and by top 50 DMRs. (PPTX 1324 kb) (PPTX 1324 kb) [file 13073_2017_419_MOESM2_ESM.pptx]

## Slide 1
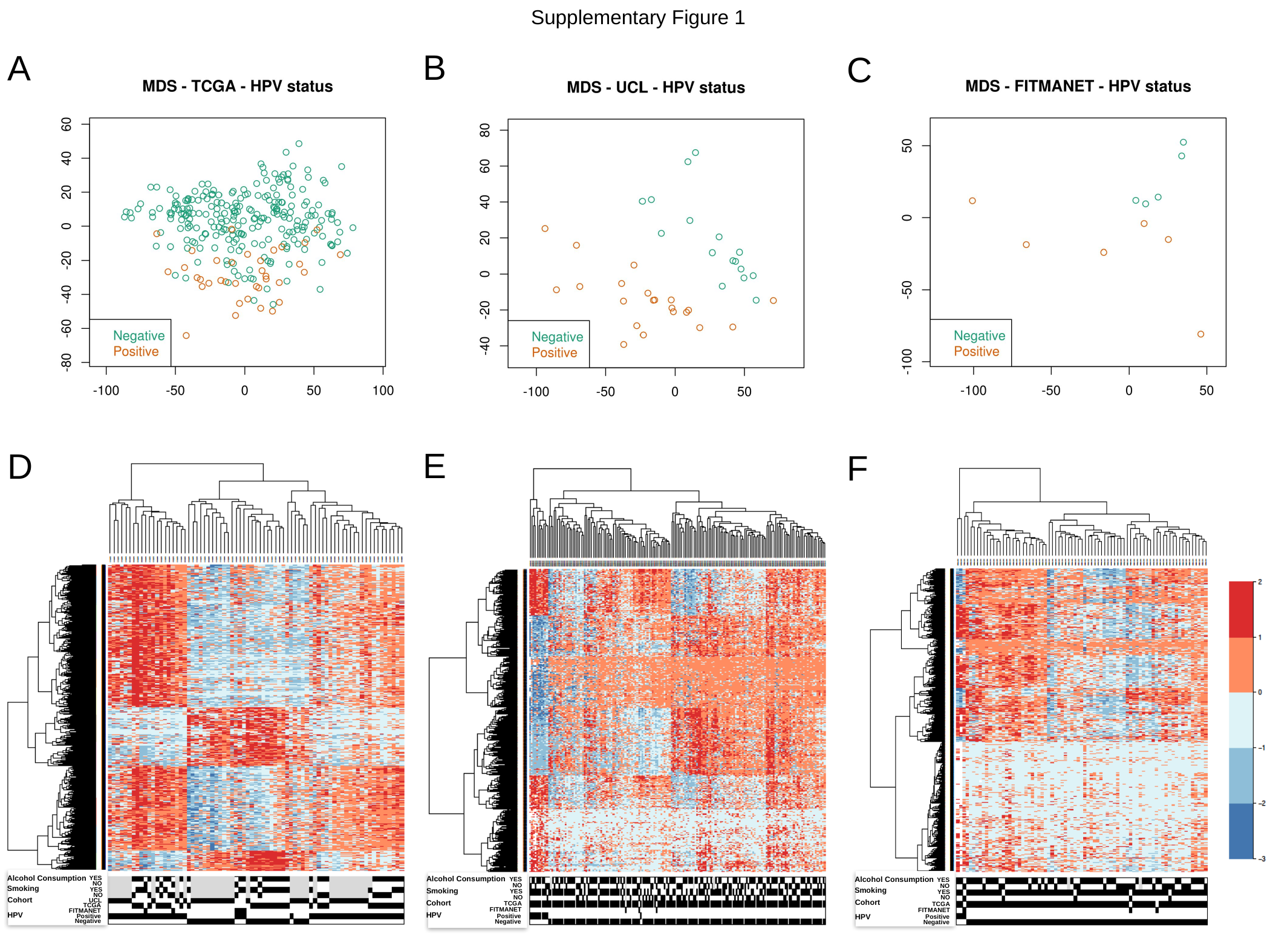

Supplementary Figure 1
B
A
C
E
D
F
Alcohol Consumption
YES
NO
Smoking
YES
NO
Cohort
UCL
TCGA
FITMANET
HPV
Positive
Negative
Alcohol Consumption
YES
NO
Smoking
YES
NO
Cohort
TCGA
FITMANET
HPV
Positive
Negative
Alcohol Consumption
YES
NO
Smoking
YES
NO
Cohort
TCGA
FITMANET
HPV
Positive
Negative

## Slide 2
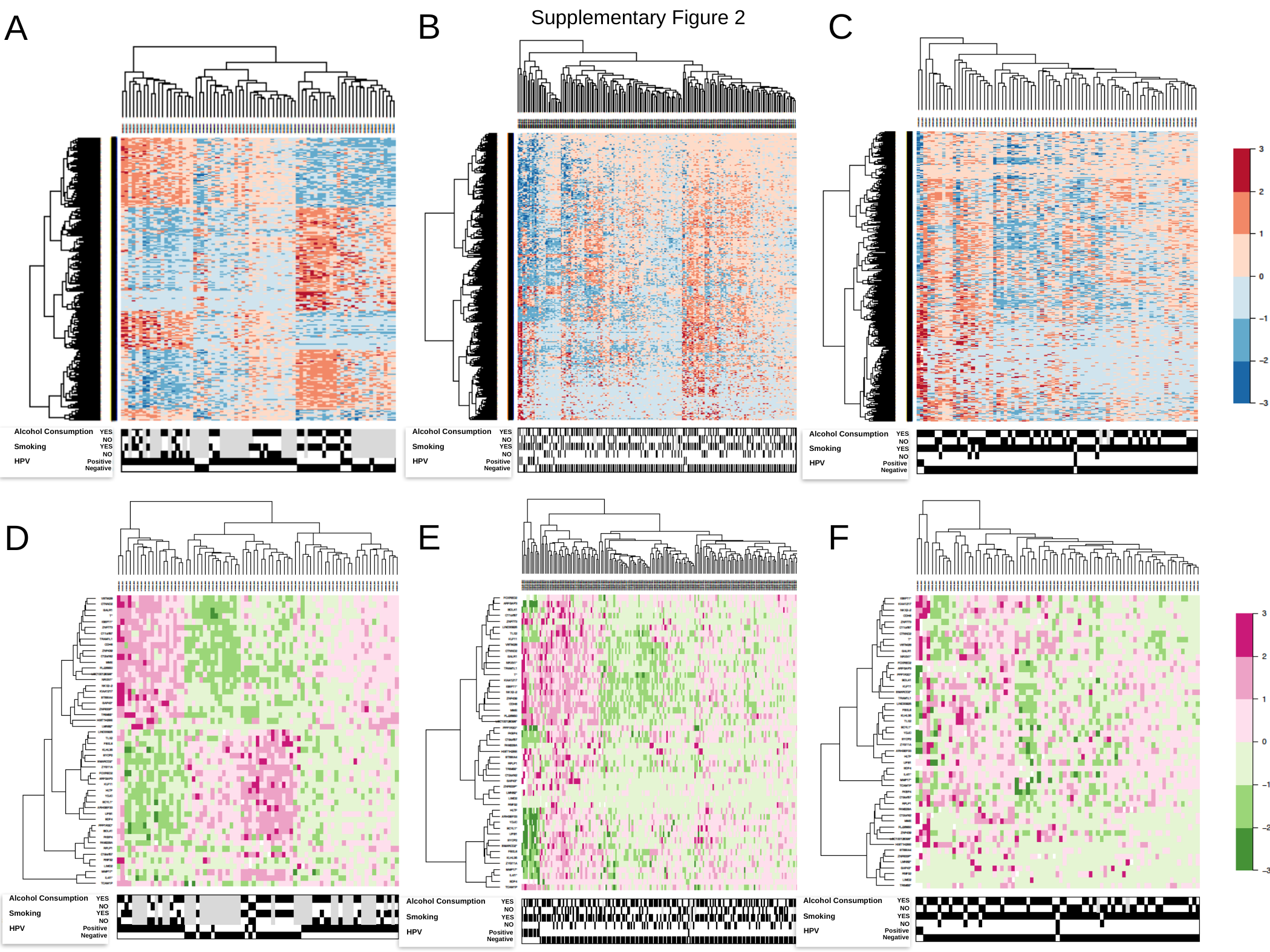

Supplementary Figure 2
B
C
A
Alcohol Consumption
YES
NO
Smoking
YES
NO
HPV
Positive
Negative
Alcohol Consumption
YES
NO
Smoking
YES
NO
HPV
Positive
Negative
Alcohol Consumption
YES
NO
Smoking
YES
NO
HPV
Positive
Negative
E
F
D
Alcohol Consumption
YES
NO
Smoking
YES
NO
HPV
Positive
Negative
Alcohol Consumption
YES
NO
Smoking
YES
NO
HPV
Positive
Negative
Alcohol Consumption
YES
NO
Smoking
YES
NO
HPV
Positive
Negative

## Slide 3
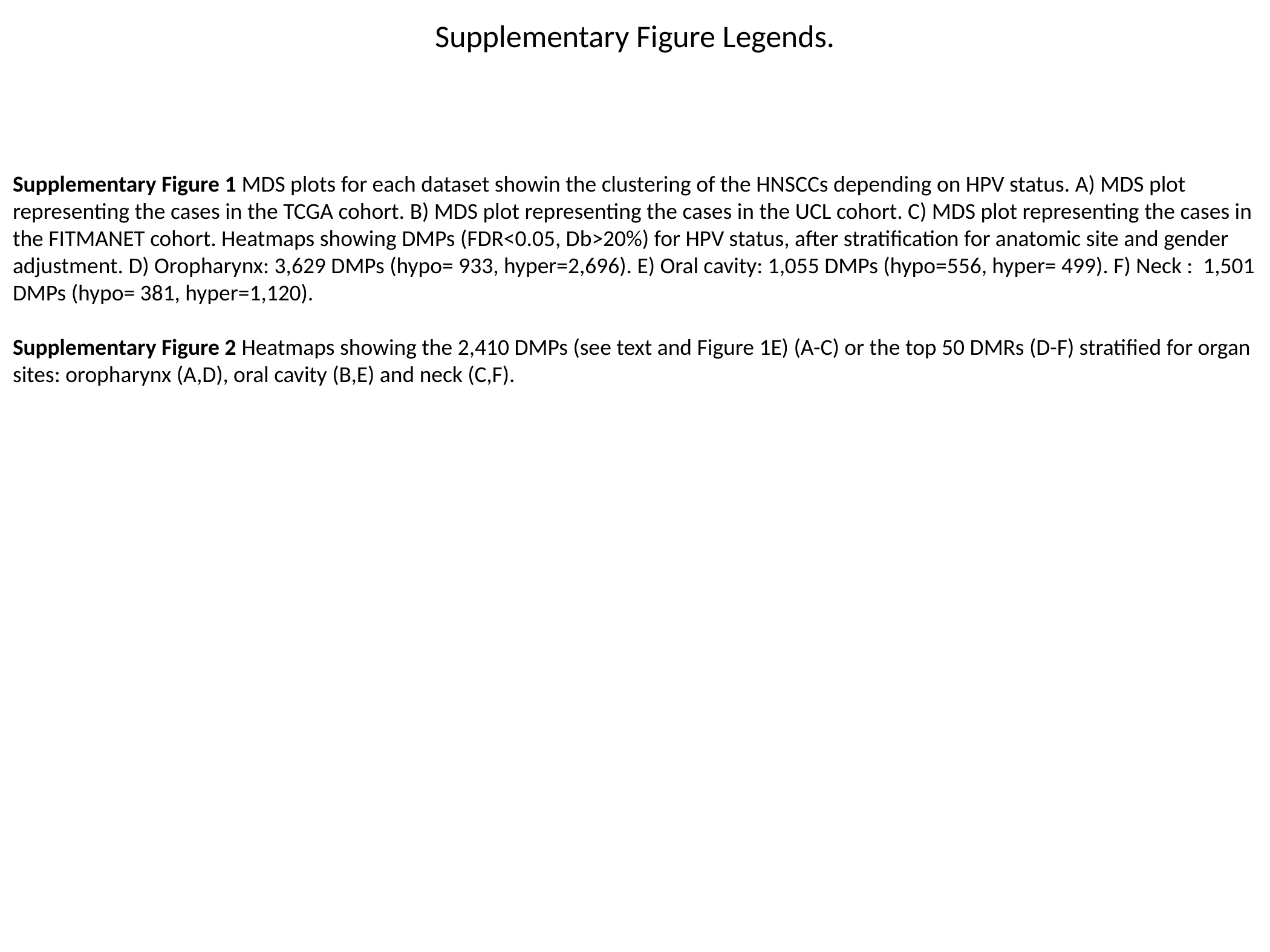

Supplementary Figure Legends.
# Supplementary Figure 1 MDS plots for each dataset showin the clustering of the HNSCCs depending on HPV status. A) MDS plot representing the cases in the TCGA cohort. B) MDS plot representing the cases in the UCL cohort. C) MDS plot representing the cases in the FITMANET cohort. Heatmaps showing DMPs (FDR<0.05, Db>20%) for HPV status, after stratification for anatomic site and gender adjustment. D) Oropharynx: 3,629 DMPs (hypo= 933, hyper=2,696). E) Oral cavity: 1,055 DMPs (hypo=556, hyper= 499). F) Neck : 1,501 DMPs (hypo= 381, hyper=1,120). Supplementary Figure 2 Heatmaps showing the 2,410 DMPs (see text and Figure 1E) (A-C) or the top 50 DMRs (D-F) stratified for organ sites: oropharynx (A,D), oral cavity (B,E) and neck (C,F).
