# Supplementary material for: Unique DNA methylation signature in HPV-positive head and neck squamous cell carcinomas
Source: Genome Med. 2017 Apr 5;9:33. doi: 10.1186/s13073-017-0419-z (PMC5382363; doi:10.1186/s13073-017-0419-z)
Supplement: Supplementary file 3 — Published methylation signatures in HNSCCs. Ad hoc pathway analysis of common hyper- and hypomethylated sites. (DOCX 29 kb) [file 13073_2017_419_MOESM3_ESM.docx]

**Additional file 3**

**Lechner-etal_signature_GenomeMedicine**

| **9 common elements in HPV(+) hypermethylated genes and HPV(+) hypermethylated genes in Degli Esposti et al** |
| --- |
| ELMO1 |
| CDH8 |
| CRMP1 |
| PCDH10 |
| MSX2 |
| SYN2 |
| PCDHB11 |
| HTR1E |
| PITX2 |

**Poage-etal_signatureCancerRes**

| **Genes whose methylation is correlated with survival (higher methylation worse survival)** | | | | | | |  |
| --- | --- | --- | --- | --- | --- | --- | --- |
|  |  |  |  |  |  |  |  |
| MESP1 |  |  |  |  |  |  |  |
| SPOCK2 |  |  |  |  |  |  |  |
| TNFAIP2 |  |  |  |  |  |  |  |
| ASB2 |  |  |  |  |  |  |  |
| TAP1 |  |  |  |  |  |  |  |
| CDKN1A |  |  |  |  |  |  |  |
| NOPE |  |  |  |  |  |  |  |
| LMO2 |  |  |  |  |  |  |  |
| CA7 |  |  |  |  |  |  |  |
| ALDH3A1 |  |  |  |  |  |  |  |
| GATM |  |  |  |  |  |  |  |
| GABRA5 |  |  |  |  |  |  |  |
|  |  |  |  |  |  |  |  |
|  |  |  |  |  |  |  |  |
| **2 common elements in "HPV+hypermethylated" and hypomethylated DMPs in Degli Esposti et al** | | | | | | | |
| TNFAIP2 |  |  |  |  |  |  |  |
| TAP1 |  |  |  |  |  |  |  |

**Parfenov-etal_signature**

| **33 common hypomethylated CpGs in HPV(+) virus-integrated(+) and hypomethylated DMPs in HPV(+) Degli Esposti et al** | | | | | | |
| --- | --- | --- | --- | --- | --- | --- |
| CpG | Gene | NearestGene |  |  |  |  |
| cg11025974 | CACNB4 | CACNB4 |  |  |  |  |
| cg08389277 | CSRNP3 | CSRNP3 |  |  |  |  |
| cg11559250 | EDIL3 | EDIL3 |  |  |  |  |
| cg03522063 | EMX2;EMX2OS | EMX2;EMX2OS |  |  |  |  |
| cg07125251 | ENPP6 | ENPP6 |  |  |  |  |
| cg12482809 | MAPRE2 | MAPRE2 |  |  |  |  |
| cg13550107 | MAPRE2 | MAPRE2 |  |  |  |  |
| cg07326648 | MAPRE2 | MAPRE2 |  |  |  |  |
| cg20396510 | MAST4 | MAST4 |  |  |  |  |
| cg10629165 | MAST4 | MAST4 |  |  |  |  |
| cg09728102 | MAST4 | MAST4 |  |  |  |  |
| cg07034329 | MAST4 | MAST4 |  |  |  |  |
| cg06373574 | NA | LOC105374988 |  |  |  |  |
| cg04392971 | NA | t-RNA-Val |  |  |  |  |
| cg07565505 | NA | IRX4 |  |  |  |  |
| cg20502003 | NA | t-RNA-Tyr |  |  |  |  |
| cg10200388 | NA | FAF1 |  |  |  |  |
| cg16082644 | NA | t-RNA-Phe |  |  |  |  |
| cg04674956 | NA | BHLHE22 |  |  |  |  |
| cg22322828 | NA | t-RNA-Tyr |  |  |  |  |
| cg01583134 | NA | t-RNA-Tyr |  |  |  |  |
| cg20932849 | NA | t-RNA-Val |  |  |  |  |
| cg19913430 | NA | t-RNA-Tyr |  |  |  |  |
| cg16039960 | NA | t-RNA-Val |  |  |  |  |
| cg24472375 | NA | NR2E1 |  |  |  |  |
| cg09537259 | NA | SESN3 |  |  |  |  |
| cg07759377 | NA | t-RNA-Tyr |  |  |  |  |
| cg02636041 | RASGEF1A | RASGEF1A |  |  |  |  |
| cg01078147 | SEMA6A | SEMA6A |  |  |  |  |
| cg25450450 | SEMA6A | SEMA6A |  |  |  |  |
| cg00410898 | STC1 | STC1 |  |  |  |  |
| cg07774884 | STC1 | STC1 |  |  |  |  |
| cg10213875 | UPP1 | UPP1 |  |  |  |  |
|  |  |  |  |  |  |  |
|  |  |  |  |  |  |  |
| **6 common hypermethylated CpGs in HPV(+) virus-integrated(+) and hypermethylated DMPs in HPV(+) Degli Esposti et al:** | | | | | | |
| CpG | Gene | NearestGene |  |  |  |  |
| cg08032619 | TBCB | TBCB |  |  |  |  |
| cg20600379 | HLA-DMB | HLA-DMB |  |  |  |  |
| cg08691577 | FAM105B | FAM105B |  |  |  |  |
| cg04307587 | ZNF576 | ZNF576 |  |  |  |  |
| cg02153747 | SVIL | SVIL |  |  |  |  |
| cg09674867 | C11orf16 | C11orf16 |  |  |  |  |

**Colacino et al Signature**

|  |  |  |  |  |  |  |  |
| --- | --- | --- | --- | --- | --- | --- | --- |
| Hypermethylated genes | Hypomethylated genes | |  |  |  |  |  |
| CCNA1 | SPDEF |  |  |  |  |  |  |
| GRB7 | RASSF1 |  |  |  |  |  |  |
| CDH11 | STAT5A |  |  |  |  |  |  |
| RUNX1T1 | MGMT |  |  |  |  |  |  |
| SYBL1 | ESR2 |  |  |  |  |  |  |
| TUSC3 | JAK3 |  |  |  |  |  |  |
|  |  |  |  |  |  |  |  |
|  |  |  |  |  |  |  |  |
|  |  |  |  |  |  |  |  |
| **2 common elements in "HPV+hypomethylated genes in Colacino et al and hypomethylated DMPs in HPV(+) in Degli Esposti et al** | | | | | | | |
| RASSF1 |  |  |  |  |  |  |  |
| STAT5A |  |  |  |  |  |  |  |
|  |  |  |  |  |  |  |  |
| **1 common elements in "HPV+hypermethylated genes in Colacino et al and hypermethylated DMPs in HPV(+) in Degli Esposti et al** | | | | | | | |
| CCNA1 |  |  |  |  |  |  |  |

**Kostareli-etal_signature**

| **Hypomethylated in HPV+** | **Hypermethylated in HPV+** | |  |  |  |
| --- | --- | --- | --- | --- | --- |
| ALDH1A2 | BDNF |  |  |  |  |
| FKBP4 | EOMES |  |  |  |  |
| GDNF | GATA4 |  |  |  |  |
| OSR2 | GFRA1 |  |  |  |  |
| PROX1 | GRIA4 |  |  |  |  |
| WIF1 | HOXA13 |  |  |  |  |
|  | IRX4 |  |  |  |  |
|  | SOX1 |  |  |  |  |
|  | TBX5 |  |  |  |  |
|  |  |  |  |  |  |
|  |  |  |  |  |  |
| **3 common elements in "HPV+hypermethylated genes in Kostareli et al and hypermethylated DMPs in HPV(+) in Degli Esposti et al** | | | | | |
| GFRA1 |  |  |  |  |  |
| IRX4 |  |  |  |  |  |
| SOX1 |  |  |  |  |  |
| **2 common elements in "HPV+hypomethylated" and hypomethylated DMPs in Degli Esposti et al** | | | | |  |
| FKBP4 |  |  |  |  |  |
| OSR2 |  |  |  |  |  |

**Lleras-etal signature**

| **Hypermethylated in HPV+** |
| --- |
| ADRA1D |
| ALX4 |
| CDKN2A |
| CTNNAL1 |
| CUTL2 |
| FBXO39 |
| GABRA4 |
| GALR1 |
| GPLD1 |
| HOXA7 |
| IGSF4 |
| IL19 |
| IPF1 |
| LOC389458 |
| MME |
| NEF3 |
| PLOD2 |
| SLC18A3 |
| SLITRK3 |
| TRAM1L1 |
| ZNF549 |
|  |
|  |
| **common elements in "HPV+hypermethylated genes in Lleras et al and hypermethylated DMPs in HPV(+) in Degli Esposti et al** |
| FBXO39 |
| GABRA4 |
| GALR1 |
| MME |
| TRAM1L1 |

**Supplementary Document 3**

**Common hypermethylated**

| **Genes commonly hypermethylated across the 3 organ groups (at gene level, not CpG level)** | | |
| --- | --- | --- |
| **Gene** | **Pathway** |  |
| TCP10 | - |  |
| ALG10 | - |  |
| C8orf87 | - |  |
| RNU6-16P | - |  |
| CTNND2 | Cadherin pathway |  |
| CDH18 | Cadherin pathway |  |
| CDH8 | Cadherin pathway |  |
| CAPS2 | Calcium signaling |  |
| CACNB2 | Calcium signaling |  |
| CALCR | Calcium signaling |  |
| PCLO | Calcium signaling |  |
| CADM1 | cell-cell adhesion in a Ca(2+)-independent manner. |  |
| CSMD3 | Complement like |  |
| CDX2 | Early embryonic development of the intestinal tract. |  |
| DNAJC6 | Endocytosis, phosphatase activity |  |
| COL6A5 | Extracell Matrix, collagen |  |
| GPC6 | Extracell Matrix; heparan sulfate proteoglycans |  |
| PREX2 | G-protein signaling family. |  |
| ST8SIA4 | Immune system |  |
| SLC52A3 | Metabolism of vitamins and cofactors. |  |
| MSX2 | Neuronal differentiation |  |
| MYT1L | Neuronal differentiation |  |
| ROBO1 | Neuronal differentiation |  |
| OTP | Neuronal differentiation |  |
| PTPRN2 | Neuronal differentiation |  |
| SORCS2 | Neuronal differentiation |  |
| RBAK-RBAKDN | Nucleic acid binding. |  |
| A2ML1 | peptidase inhibitor activity. |  |
| KCNA1 | potassium channel activity. |  |
| FRMD4A | protein binding, bridging. |  |
| TRIM55 | protein-protein interactions |  |
| KIAA1217 | Required for normal development of intervertebral disks. |  |
| ADARB2 | RNA editing |  |
| ZBED9 | Zinc Finger, nucleic acid binding |  |
| ZNF304 | Zinc Finger, nucleic acid binding |  |
| ZNF235 | Zinc Finger, nucleic acid binding |  |
|  |  |  |
|  |  |  |
|  | Cadherin pathway, Ca2+ signaling, neuronal differentiation |  |

Common hypomethylated

| **Genes commonly hypomethylated across the 3 organ groups (at gene level, not CpG level)** | | | | | | |
| --- | --- | --- | --- | --- | --- | --- |
| **Gene** | **Pathway** |  |  |  |  |  |
| BOLA1 | - |  |  |  |  |  |
| KLHL35 | - |  |  |  |  |  |
| C10orf88 | - |  |  |  |  |  |
| STK3 | Apoptosis |  |  |  |  |  |
| CARD9 | Apoptosis |  |  |  |  |  |
| NTN1 | Apoptosis |  |  |  |  |  |
| RAD9B | Cell cycle |  |  |  |  |  |
| SMC1B | Cell cycle |  |  |  |  |  |
| SEH1L | cell cycle |  |  |  |  |  |
| CDC42EP5 | Cell division, G-protein signaling family. | | | |  |  |
| YPEL1 | Cell division. | |  |  |  |  |
| COX6A2 | cytochrome-c oxidase activity | | |  |  |  |
| OTUD7A | Deubiquitinating enzymes | |  |  |  |  |
| B3GALT6 | extracellular matrix, glycosaminoglycan synthesis. | | | |  |  |
| MMP17 | extracellular matrix, metallo protein | | |  |  |  |
| ARFGAP3 | G-protein signaling family. | | |  |  |  |
| RGS14 | G-protein signaling family. | | |  |  |  |
| YDJC | hydrolase activity, | |  |  |  |  |
| HIF1AN | hypoxia, oxidative stress | |  |  |  |  |
| LOC729683 | ncRNAs |  |  |  |  |  |
| C7orf13 | ncRNAs |  |  |  |  |  |
| LINC00239 | ncRNAs |  |  |  |  |  |
| DLEU1 | ncRNAs, Delated in leukemia | | |  |  |  |
| LINC00925 | ncRNAs, MIR9-3 Host Gene | | |  |  |  |
| MIR9-3 | ncRNAs, MIR9-3 Host Gene, involved in leukemia | | | |  |  |
| ABCA17P | ncRNAs, pseudogenes | |  |  |  |  |
| IRX3 | Neuronal differentiation | |  |  |  |  |
| SYNGR3 | Neuronal differentiation | |  |  |  |  |
| CCDC177 | Neuronal differentiation | |  |  |  |  |
| GJC2 | Neuronal differentiation | |  |  |  |  |
| FOXRED2 | Oxidoreductase | |  |  |  |  |
| IL4I1 | Oxidoreductase | |  |  |  |  |
| KCNQ1 | Potassium Channel | |  |  |  |  |
| TBCD | protein folding | |  |  |  |  |
| MDGA1 | Putative Neuronal differentiation | | |  |  |  |
| RPL31 | ribosomal protein | |  |  |  |  |
| TLX2 | t-cell leukemia related, neural differentiation | | | |  |  |
| ADNP | Transcriptional factor | |  |  |  |  |
| SOX30 | Transcriptional factor | |  |  |  |  |
| ISL2 | Transcriptional factor | |  |  |  |  |
| FZD5 | Wnt signaling proteins | |  |  |  |  |
| ZNF541 | Zinc Finger, nucleic acid binding | | |  |  |  |
| ZNF280D | Zinc Finger, nucleic acid binding | | |  |  |  |
|  |  |  |  |  |  |  |
| Apoptosis, Cell Cycle, ncRNAs | | |  |  |  |  |
