# Supplementary material for: Unique DNA methylation signature in HPV-positive head and neck squamous cell carcinomas
Source: Genome Med. 2017 Apr 5;9:33. doi: 10.1186/s13073-017-0419-z (PMC5382363; doi:10.1186/s13073-017-0419-z)
Supplement: Supplementary file 9 — Co-methylation analysis. (PPTX 3382 kb) [file 13073_2017_419_MOESM9_ESM.pptx]

## Slide 1
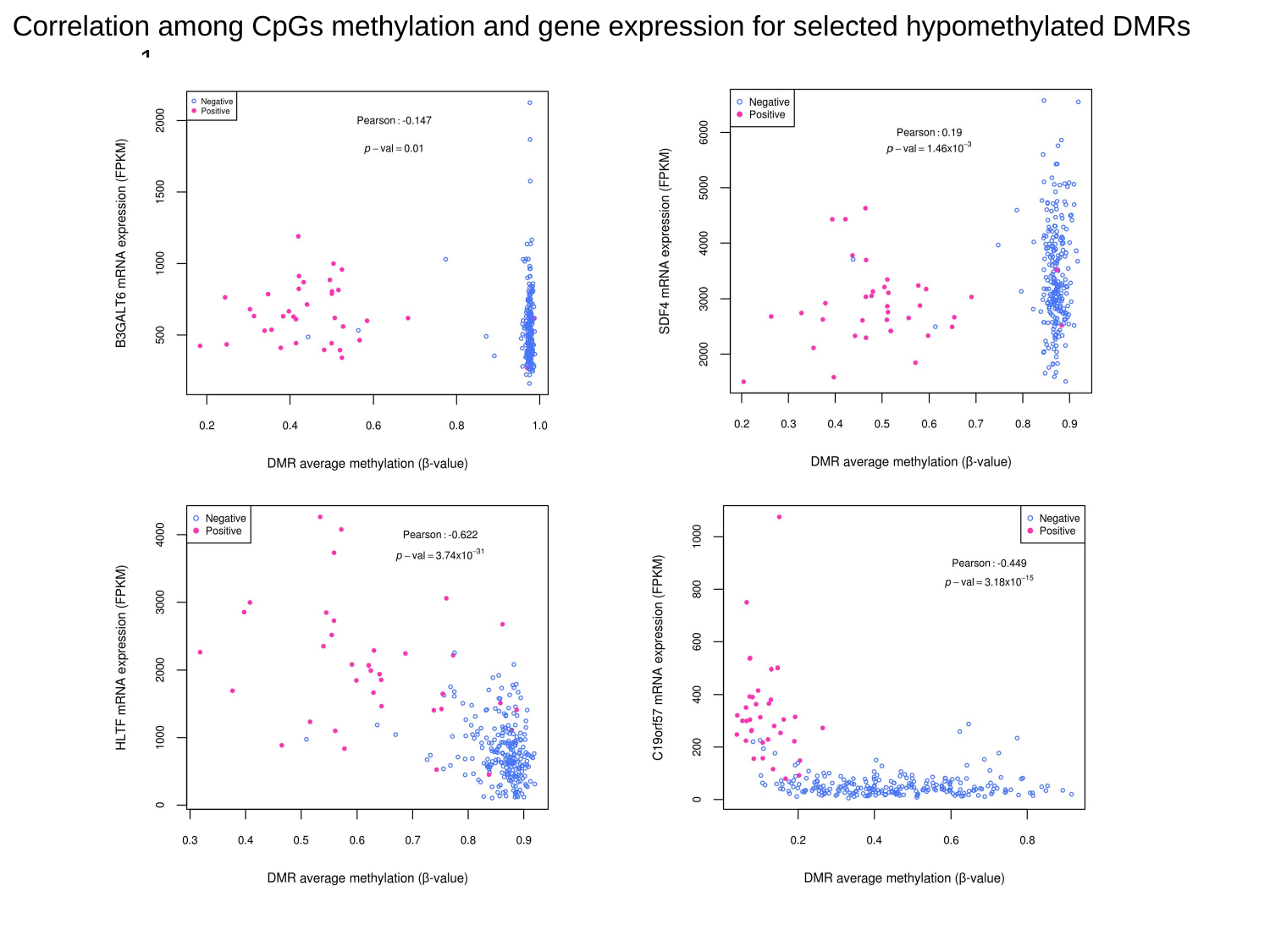

Correlation among CpGs methylation and gene expression for selected hypomethylated DMRs 	1

## Slide 2
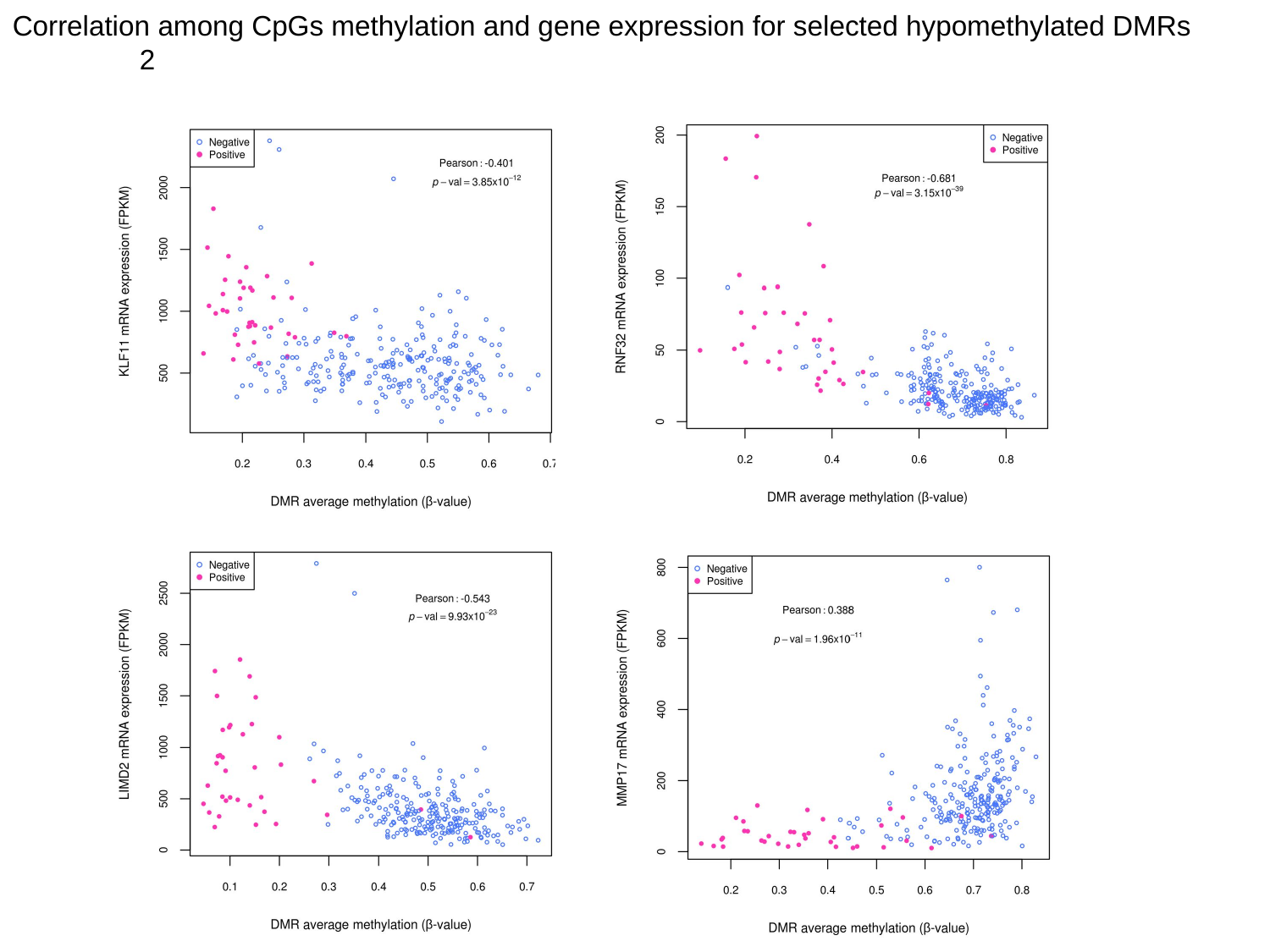

Correlation among CpGs methylation and gene expression for selected hypomethylated DMRs 	2

## Slide 3
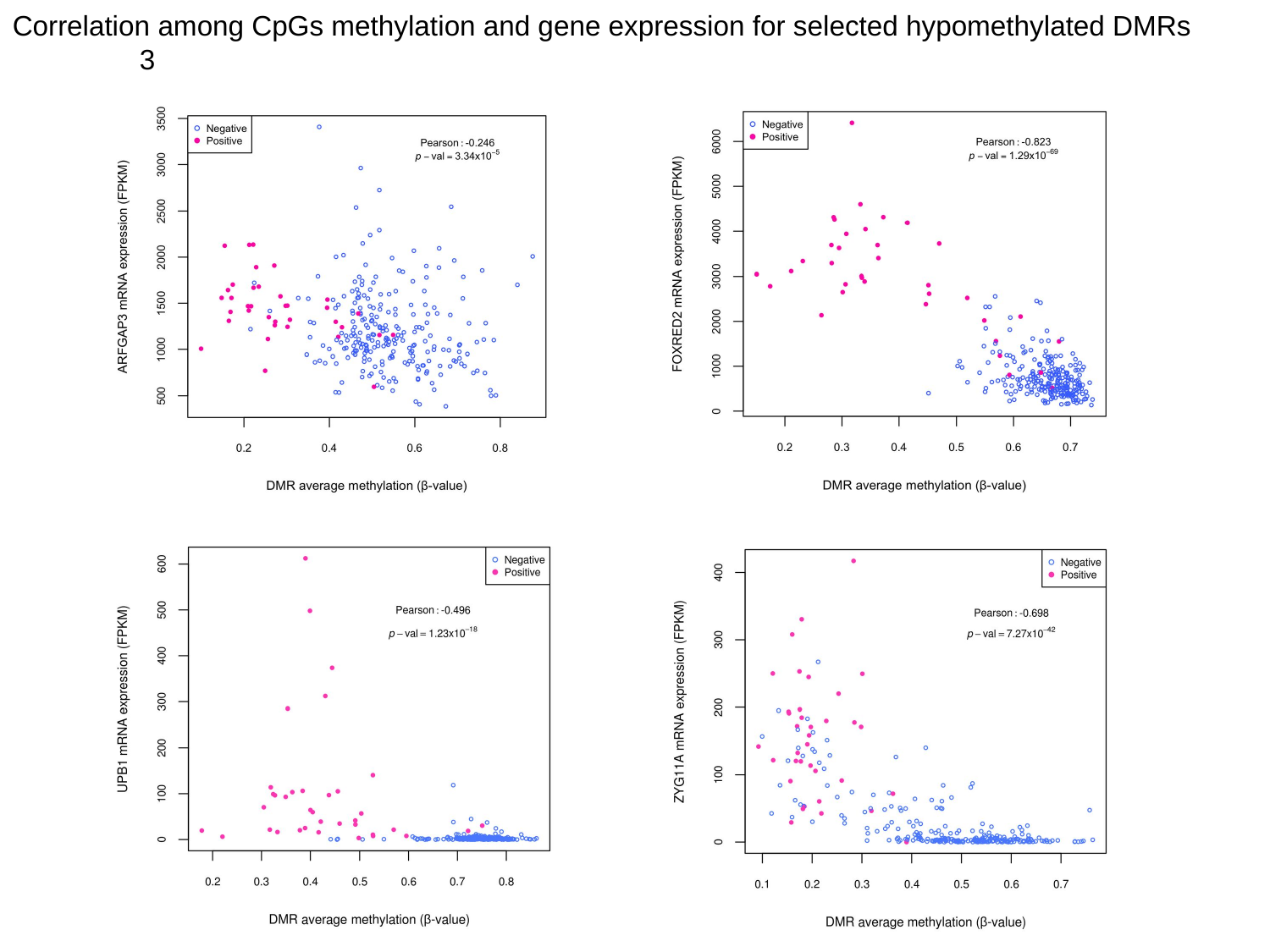

Correlation among CpGs methylation and gene expression for selected hypomethylated DMRs 	3

## Slide 4
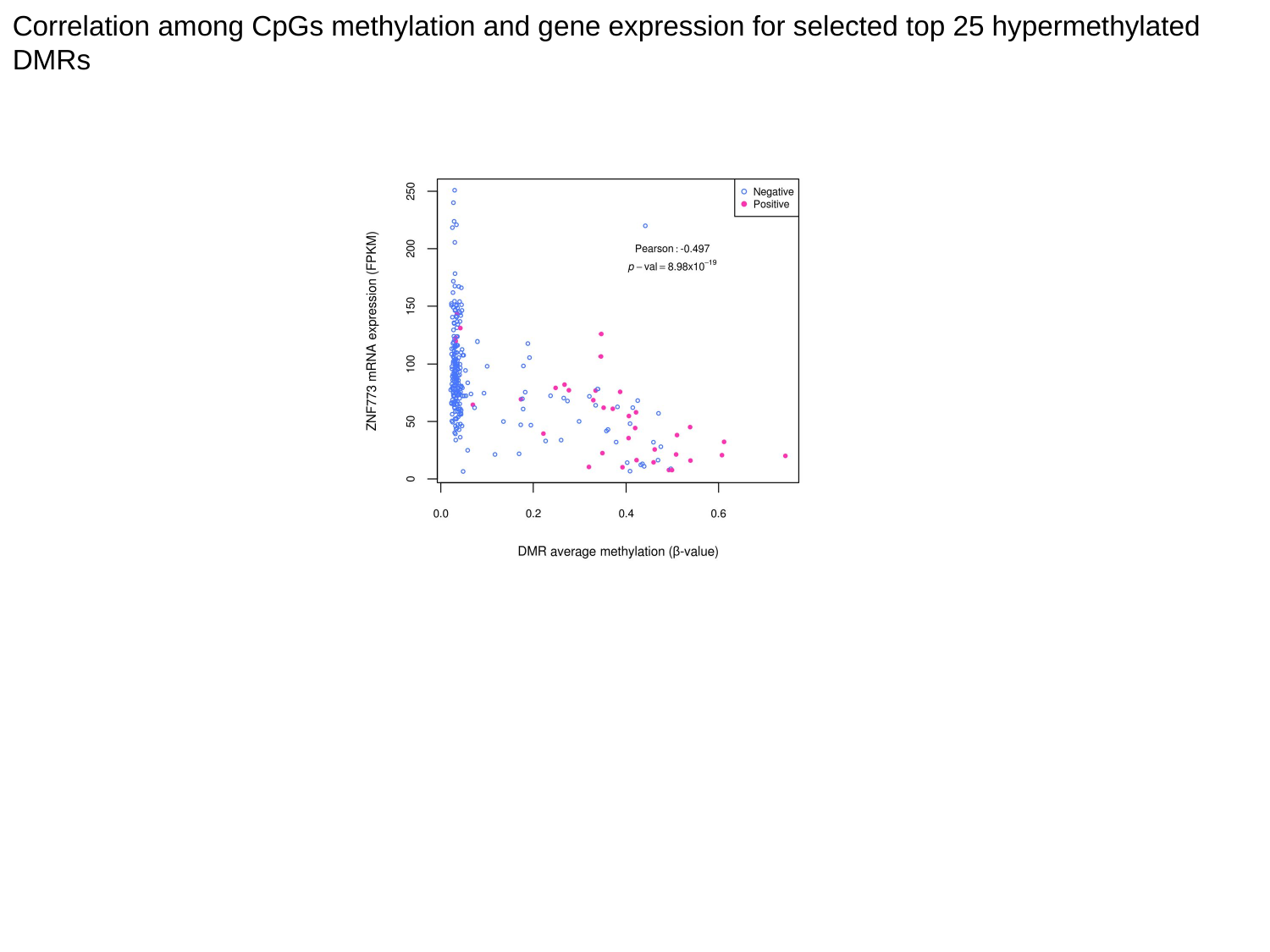

Correlation among CpGs methylation and gene expression for selected top 25 hypermethylated DMRs

## Slide 5
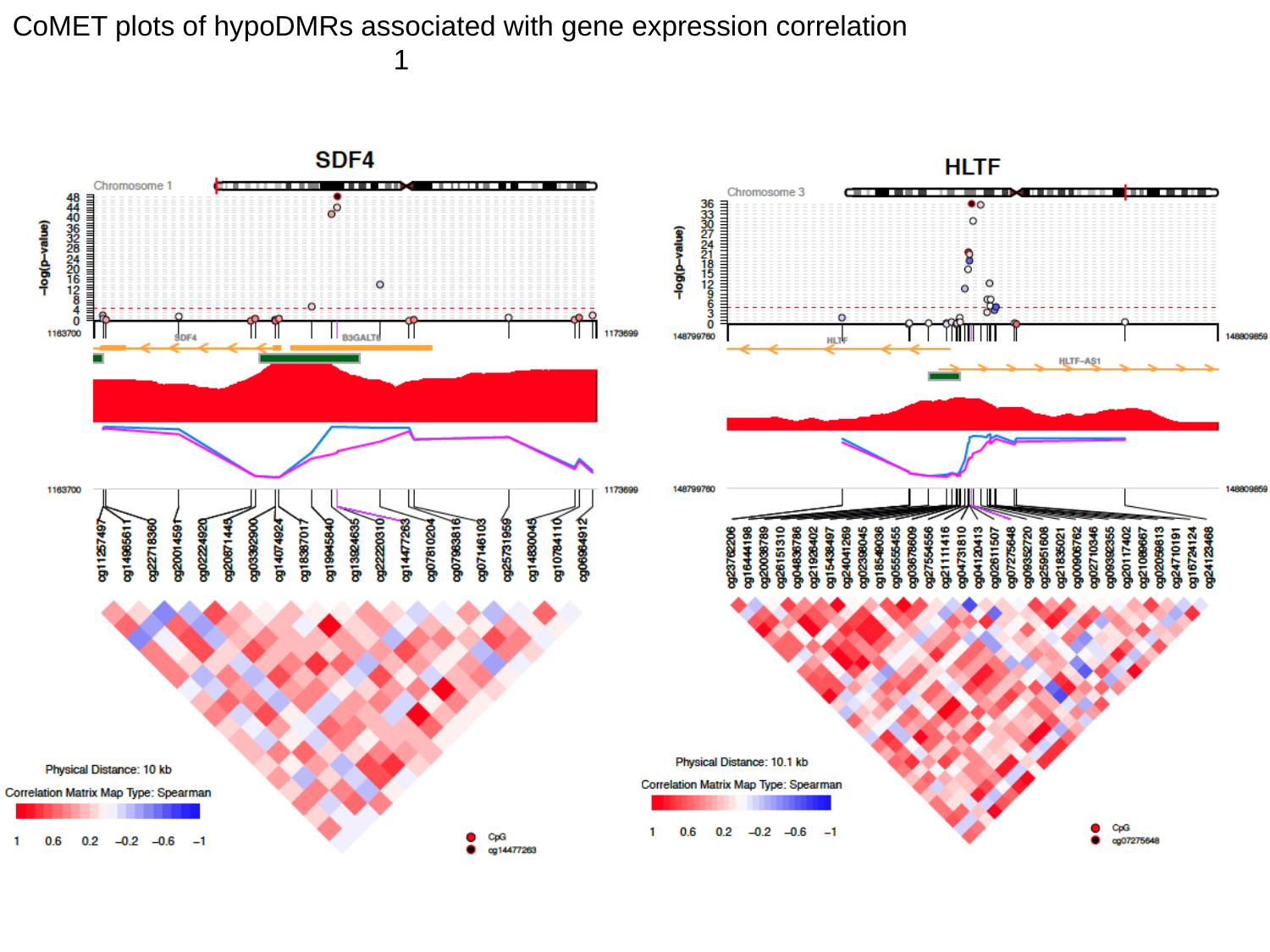

CoMET plots of hypoDMRs associated with gene expression correlation					1

## Slide 6
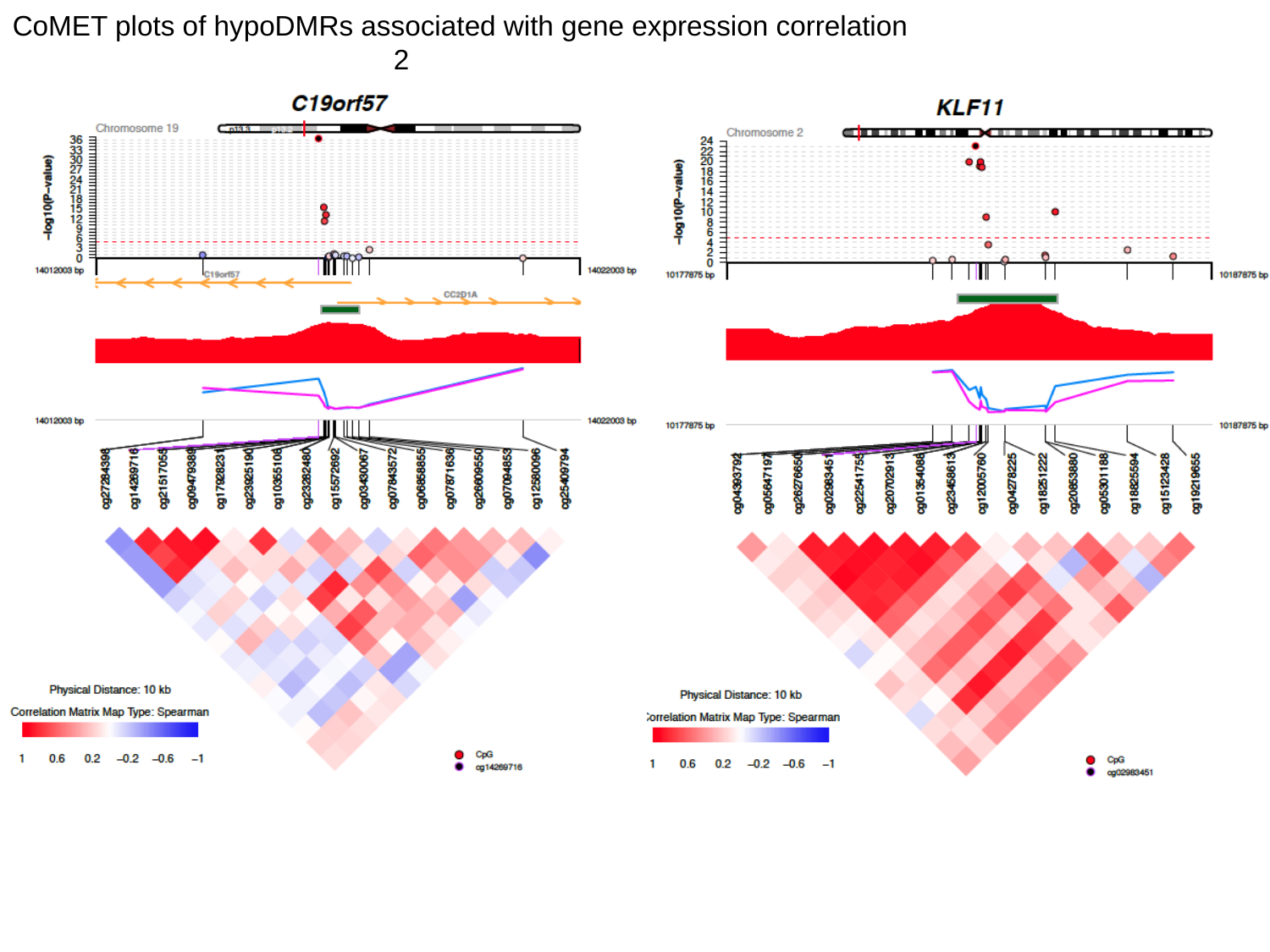

CoMET plots of hypoDMRs associated with gene expression correlation					2

## Slide 7
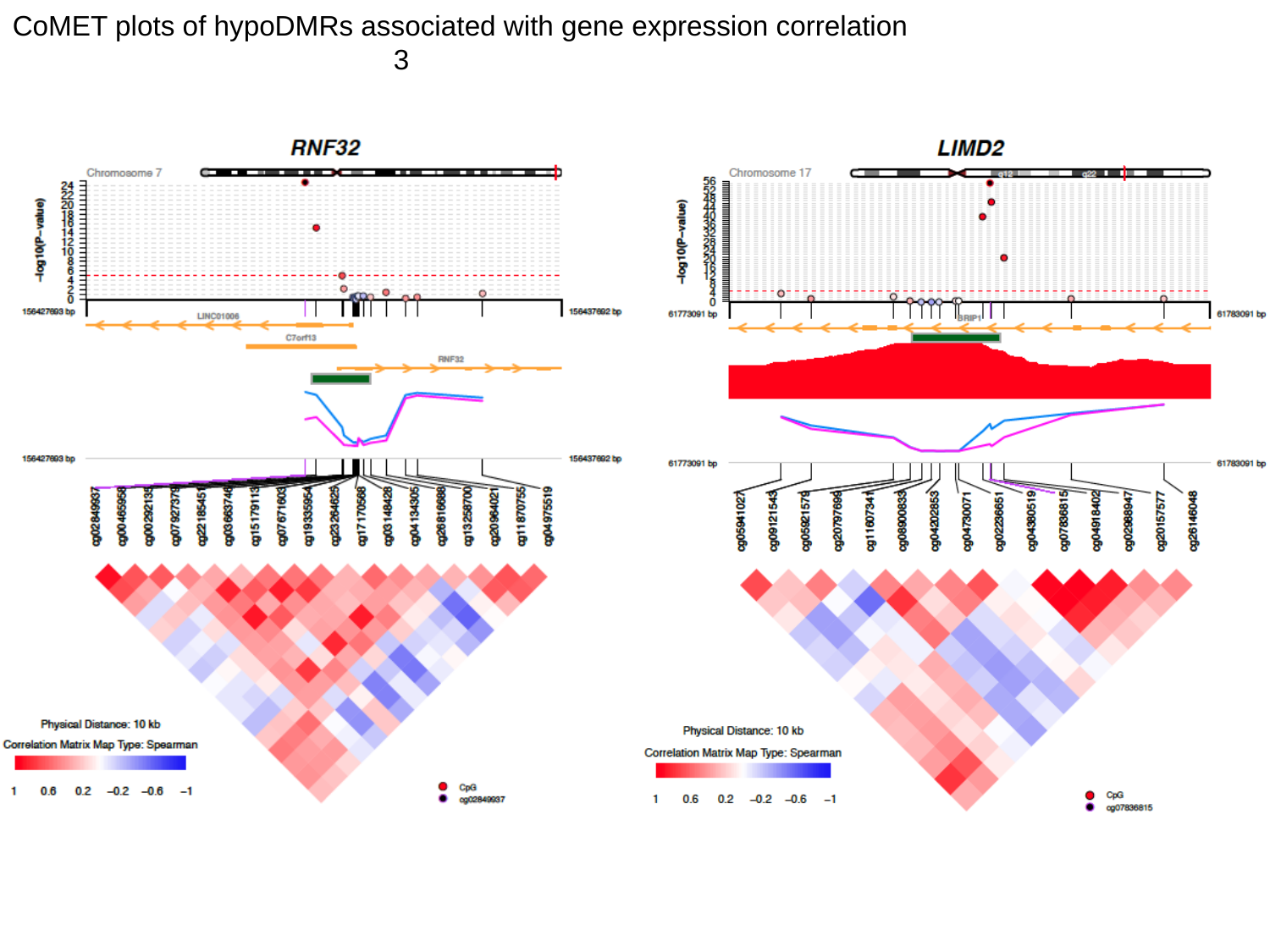

CoMET plots of hypoDMRs associated with gene expression correlation					3

## Slide 8
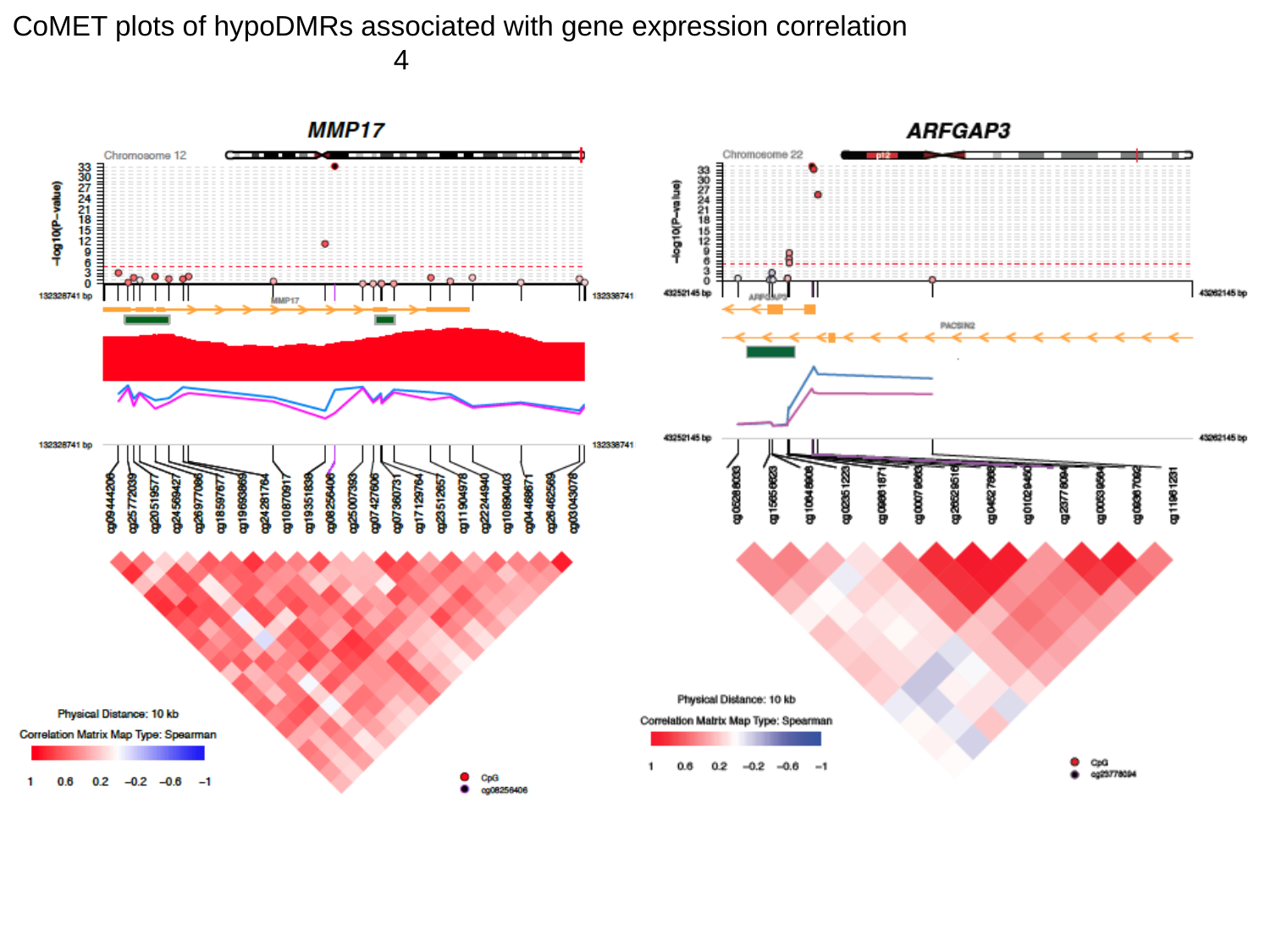

CoMET plots of hypoDMRs associated with gene expression correlation					4

## Slide 9
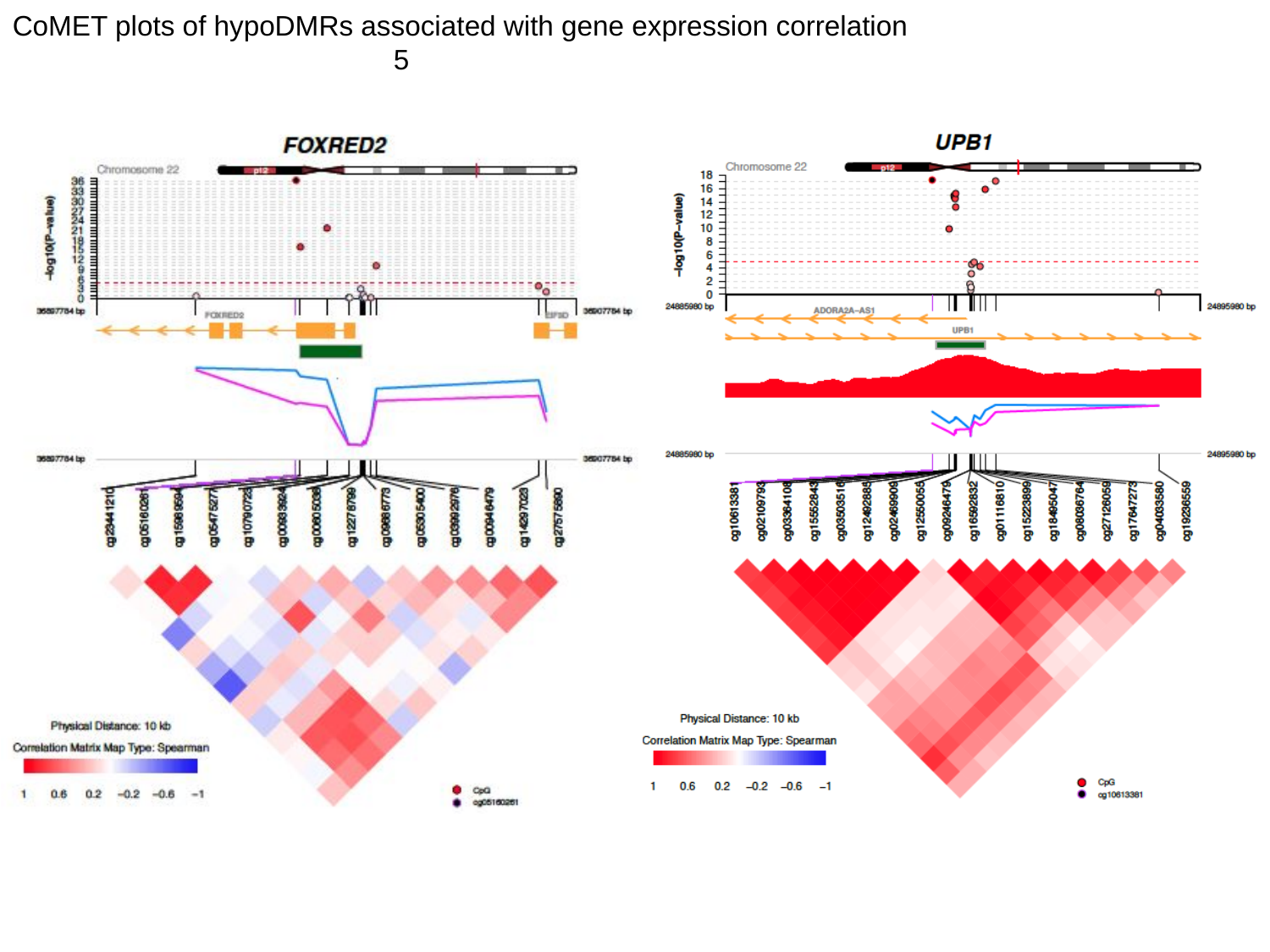

CoMET plots of hypoDMRs associated with gene expression correlation					5

## Slide 10
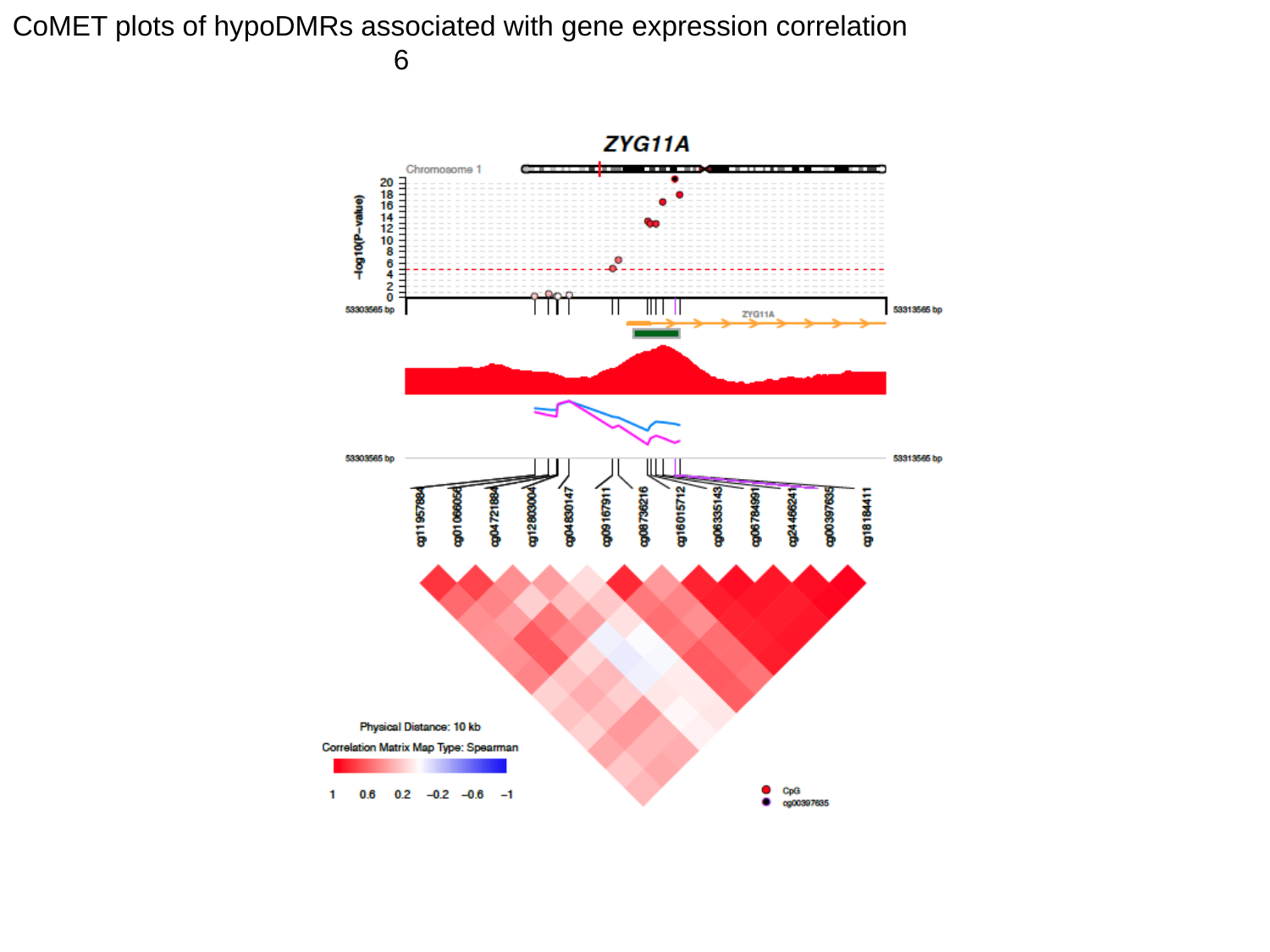

CoMET plots of hypoDMRs associated with gene expression correlation					6

## Slide 11
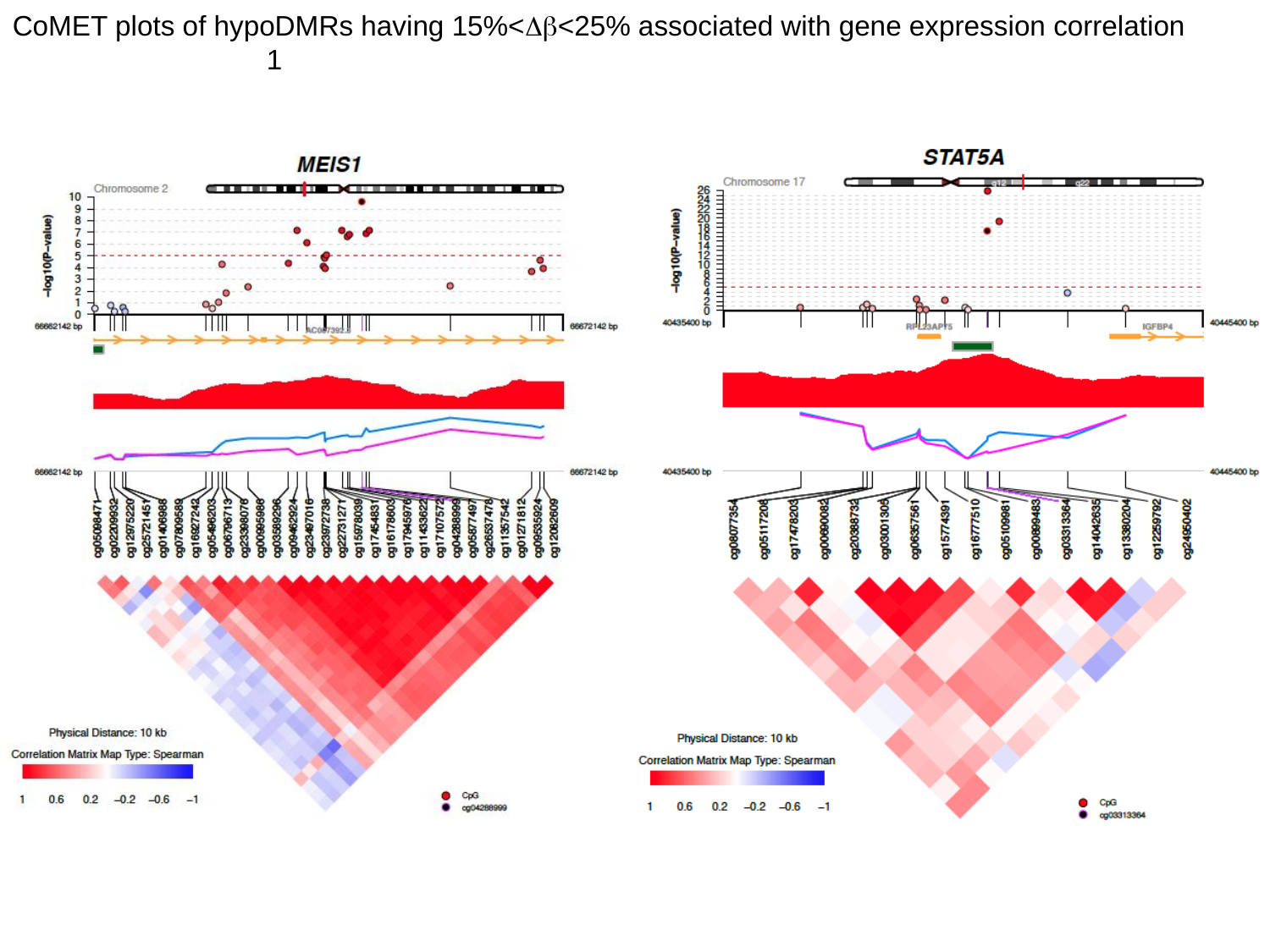

CoMET plots of hypoDMRs having 15%<Db<25% associated with gene expression correlation		1
